# Influence of peer networks on physician adoption of new drugs

**DOI:** 10.1371/journal.pone.0204826

**Published:** 2018-10-01

**Authors:** Julie M. Donohue, Hasan Guclu, Walid F. Gellad, Chung-Chou H. Chang, Haiden A. Huskamp, Niteesh K. Choudhry, Ruoxin Zhang, Wei-Hsuan Lo-Ciganic, Stefanie P. Junker, Timothy Anderson, Seth Richards-Shubik

**Affiliations:** 1 Department of Health Policy and Management, Graduate School of Public Health, University of Pittsburgh, Pittsburgh, Pennsylvania, United States of America; 2 Department of Statistics, School of Engineering and Natural Sciences, Istanbul Medeniyet University, Istanbul, Turkey; 3 Department of Biostatistics and Medical Informatics, School of Medicine, Istanbul Medeniyet University, Istanbul, Turkey; 4 Center for Pharmaceutical, Policy and Prescribing, Health Policy Institute, University of Pittsburgh, Pittsburgh, Pennsylvania, United States of America; 5 Department of Medicine, School of Medicine, University of Pittsburgh, Pittsburgh, Pennsylvania, United States of America; 6 Center for Health Equity Research and Promotion, Veterans Affairs Pittsburgh Healthcare System, Pittsburgh, Pennsylvania, United States of America; 7 Department of Biostatistics, Graduate School of Public Health, University of Pittsburgh, Pittsburgh, Pennsylvania, United States of America; 8 Department of Health Care Policy, Harvard Medical School, Harvard University, Boston, Massachusetts, United States of America; 9 Department of Medicine, Division of Pharmacoepidemiology and Pharmacoeconomics, Brigham and Women’s Hospital and Harvard Medical School, Boston, Massachusetts, United States of America; 10 Quality and Operations Support, The Permanente Medical Group, Inc., Oakland, California, United States of America; 11 Department of Pharmacy, Practice and Science, College of Pharmacy, University of Arizona, Tucson, Arizona, United States of America; 12 Department of Medicine, University of California San Francisco, San Francisco, California, United States of America; 13 Department of Economics, College of Business and Economics, Lehigh University, Bethlehem, Pennsylvania, United States of America; Dartmouth-Hitchcock Medical Center, UNITED STATES

## Abstract

Although physicians learn about new medical technologies from their peers, the magnitude and source of peer influence is unknown. We estimate the effect of peer adoption of three first-in-class medications (dabigatran, sitigliptin, and aliskiren) on physicians’ own adoption of those medications. We included 11,958 physicians in Pennsylvania prescribing anticoagulant, antidiabetic, and antihypertensive medications. We constructed 4 types of peer networks based on shared Medicare and Medicaid patients, medical group affiliation, hospital affiliation, and medical school/residency training. Instrumental variables analysis was used to estimate the causal effect of peer adoption (fraction of peers in each network adopting the new drug) on physician adoption (prescribing at least the median number prescriptions within 15 months of the new drug’s introduction). We illustrate how physician network position can inform targeting of interventions to physicians by computing a social multiplier. Dabigatran was adopted by 25.2%, sitagliptin by 24.5% and aliskiren by 8.3% of physicians. A 10-percentage point increase in peer adoption in the patient-sharing network led to a 5.90% (SE = 1.50%, p<0.001) increase in physician adoption of dabigatran, 8.32% (SE = 1.51%, p<0.001) increase in sitagliptin, and 7.84% increase in aliskiren adoption (SE = 2.93%, p<0.001). Peer effects through shared hospital affiliation were positive but not significant, and medical group and training network effects were not reliably estimated. Physicians in the top decile of patient-sharing network peers were estimated to have nearly 2-fold stronger influence on their peers’ adoption compared to physicians in the top decile of prescribing volume. Limitations include lack of detailed clinical information and pharmaceutical promotion, variables which may influence physician adoption but which are unlikely to bias our peer effect estimates. Peer adoption, especially by those with whom physicians share patients, strongly influenced physician adoption of new drugs. Our study shows the potential for using information on physician peer networks to improve technology diffusion.

## Introduction

Diffusion of technology in US healthcare, while influenced partly by payer policies regarding coverage and reimbursement, is to a large extent driven by the individual decisions of practicing physicians. The relative absence of centralized technology assessment in the US reflects the importance of physician autonomy, allows for flexible responses to rapidly changing evidence, and creates opportunities to tailor decisions to individual patients. However, it comes at the cost of wide geographic variation,[1, 2] excess spending, and sluggish translation of evidence into practice.[3, 4] Recognizing that no US regions provide uniformly better care, an Institute of Medicine report recommended that efforts to achieve high-value healthcare target the loci of decision-making–hospitals, physician groups, and individual providers[5]. Yet the scale of changing provider behavior at the individual-level is daunting.

To make the most efficient use of resources for educating the workforce,[6–8] providers may be viewed as embedded in social systems.[9–11] While it is known that physicians learn from each other, the magnitude of peer influence is poorly understood and is a largely untapped resource. The tools of social network analysis can be used to map the connections among providers, and identify those playing a central role among their peers.[12] The extant studies applying social network methods to technology diffusion among physicians,[11, 13–22] have been limited to uptake of a single technology, and small physician samples.[16, 21, 23, 24] Prior studies have relied primarily on physicians’ self-reported information on peer connections—information that, while informative, would be cost-prohibitive to collect on a large scale.

We take advantage of increasingly large and detailed healthcare databases to examine the value of harnessing social network information to drive physician adoption of evidence-based technologies. First, we constructed peer networks among nearly 12,000 physicians drawing on multiple sources of information to form peer networks based on shared patients, practice settings, and training. Second, we estimated the magnitude of peer influence on technology adoption using as natural experiments the introductions of three new prescription drugs varying in novelty, clinical indication, number of competitors, and the specialties of physicians prescribing them. Third, we illustrate how simple information on physicians’ positions in their peer networks can be used to target interventions to change physician behavior.

## Methods

### Study setting and data sources

Our study setting was Pennsylvania, the 5^th^ largest US state, the population of which mirrors national averages in socio-demographic characteristics and on measures of health care utilization.[25] The study period during which the three drugs of interest were introduced and over which we measured their adoption was 2007–2011. We obtained 5 data sources all of which contained and were linked by National Provider Identifier (NPI). First, physician-level prescribing data were obtained from QuintilesIMS’s Xponent^™^ database which directly captures >70% of all US prescriptions and uses a projection method to represent all retail prescriptions filled. Second, from the American Medical Association (AMA) Masterfile we obtained information on physician characteristics including demographics (age, sex), and training (e.g., medical school, graduation year). Third, we obtained information on physician’s organizational affiliations from QuintilesIMS’s Healthcare Organizational Services (HCOS^™^) database which captures provider affiliations with >29,000 US practices, clinics, and hospitals. Fourth, we obtained administrative claims data on all (fee-for-service and managed care) enrollees in PA Medicaid from the PA Department of Human Services. Fifth, Medicare data for all fee-for-service enrollees with Part D pharmacy benefits were obtained from the Centers for Medicare and Medicaid Services (CMS). Medicaid and Medicare cover approximately 1/3 of Pennsylvanians.[26]

### New medications of interest

We measured physician adoption of dabigatran (an oral anticoagulant initially approved to treat atrial fibrillation on 10/19/2010), sitagliptin (an oral dipeptidyl peptidase-4 inhibitor approved to treat diabetes on 10/16/2006), and aliskiren (an oral direct renin inhibitor approved to treat hypertension on 3/05/2007) (S1 Supporting Information). All three medications were first-in-class, with a novel mechanism of action, although they varied in the extent to which they were viewed as superior within the broader therapeutic class,[27–30] the relevant patient populations, and the availability of substitutes.

### Physician cohorts

We constructed three cohorts of physicians applying 4 broad inclusion criteria. We required physicians to: a) prescribe medications in one or more drug classes of interest (oral anticoagulant, antidiabetic or antihypertensive medications) during the study period (**Parts a-c of** S1 Table); b) have an AMA Masterfile record and a Pennsylvania practice address; c) have a record in HCOS database; and d) demonstrate some minimal prescribing in the therapeutic class in the first 15 months after the new drug was introduced (minimal defined as ≥1 prescription/quarter) (S1–S3 Figs **for cohort construction)**.

### Peer network construction

**Fig 1:**
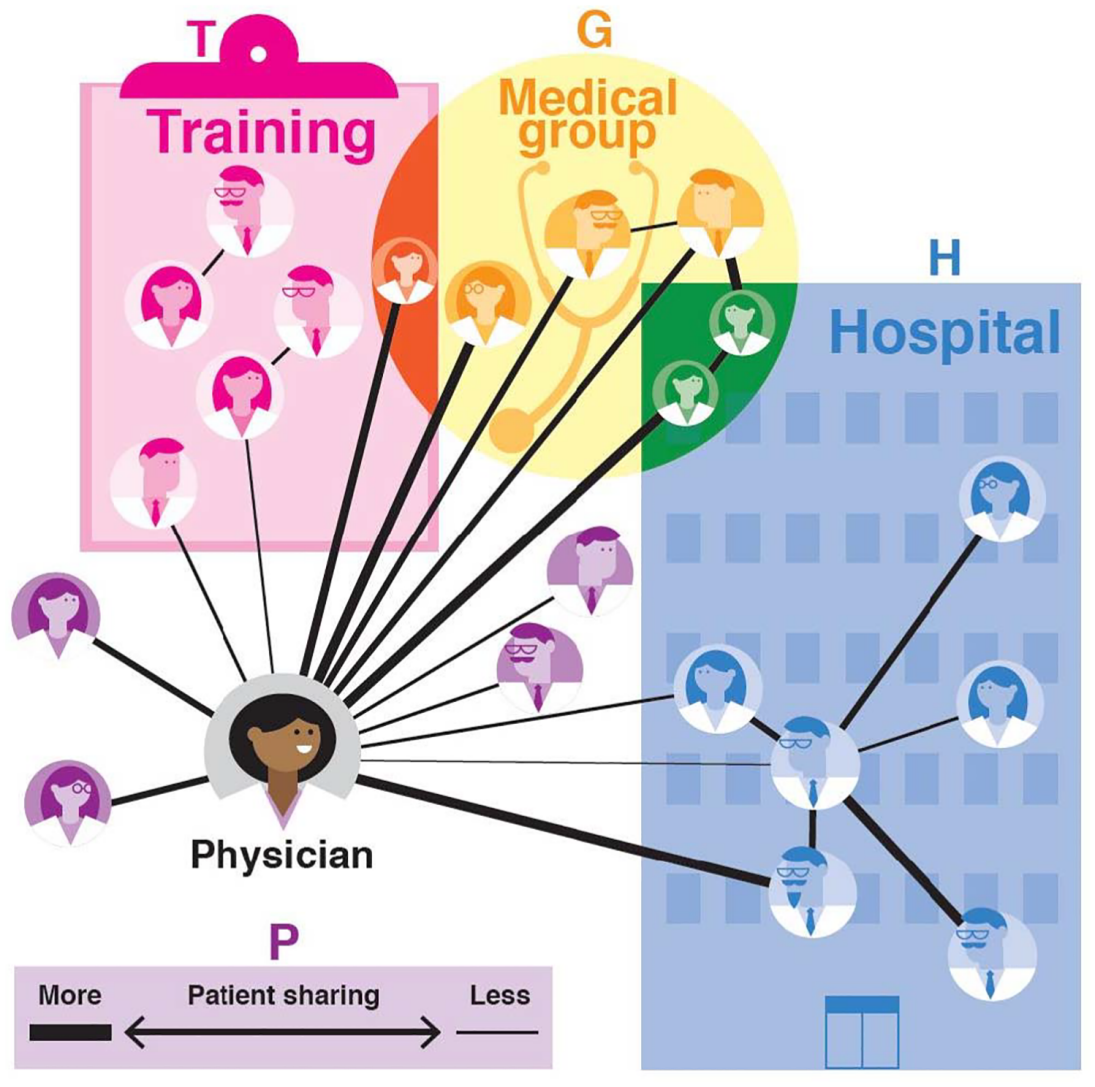
Illustration of physician peer networks. The figure shows the affiliation networks of a typical physician in our study cohorts (bottom left). Within the cohort of physicians prescribing the drug class of interest, she is connected to peers (shown in pink) with whom she attended the same medical school (one year +/-) or with whom she completed the same residency program (one year +/-). She is connected to peers (shown in yellow) through the medical group where she has an outpatient practice and to peers (shown in blue) through the hospital where she admits patients. In addition, she shares Medicare and Medicaid patients with several physicians. The patient-sharing network is represented by the lines in the figure; line thickness corresponds to the number of patients shared between physicians. Connections shown in orange are affiliated with the physician in this illustration through shared training institution and medical group. Connections shown in green share a medical group and hospital affiliation in common with the physician. Connections shown in purple only have shared patients with the physician.

Physicians form relationships with peers whom they meet during training, and in office- and hospital-based practice settings, and form referral and information networks with peers both within and outside of their own health systems.[11, 14, 15, 31–35] To capture these rich, and potentially overlapping peer networks we formed 4 types of physician social networks illustrated in Fig 1. We used the network analysis library Igraph in python[36] to construct the networks, and measure network characteristics. Network construction is briefly summarized here with additional information provided in S1 Supporting Information.

First, we constructed *patient-sharing networks* (*P*) (depicted by black lines in Fig 1) using a previously published approach developed using patient-sharing in Medicare data.[37–39] Barnett and colleagues used self-reported connections to validate claims-based connections, reporting that two physicians billing Medicare for at least 9–10 patients in common during the same year were highly likely to self-report having a relationship based on referrals and/or advice.[37] We extend prior studies using data from a single payer to construct physician networks[40–43] by combining Medicare *and* Medicaid claims for unique patients to form the patient-sharing network. Second, we constructed a *medical group network* (*G)* using data from HCOS. We identified as medical group peers all physicians in the prescribing cohort with whom a physician shared a medical group or clinic affiliation. Third, a *hospital network* (*H*) was constructed similarly using data from HCOS on shared hospital affiliation (e.g., attending or admitting); we include in the hospital network all physicians in the cohort with a shared hospital affiliation. Last, a *training network* (*T*) was constructed using AMA Masterfile data on institutions attended and dates of graduation. Two physicians were connected if they attended the same medical school or the same residency program within +/-1 year of each other.

### Measuring adoption

We defined adoption as writing at least the median number of prescriptions among physicians prescribing the new drug at least once in its first 15 months on the market. The medians were 7, 13, and 7 prescriptions for dabigatran, sitagliptin, and aliskiren, respectively. We tested the robustness of our findings to alternative specifications of the adoption measure (e.g., > = 1, > = 15) (S12 Table).

*Peer adoption* in each network was the key independent variable in our analysis and was measured as the fraction of a physician’s peers who adopted the new drug (i.e., who had at least the median number of prescriptions in the first 15 months the new drug was on the market). This peer adoption measure (the “peer adoption rate”) was computed in each of the four networks separately as we hypothesized each to have a distinct influence. Hence, there are four separate adoption variables among peers in the: (1) patient-sharing network, (2) medical group network, (3) hospital network, and (4) training network. Peers in the training, medical group and hospital networks were assumed to have equal influence, while peers in the patient-sharing network were weighted based on the number of patients shared (i.e., a weighted average was used to compute the adoption rate in the patient-sharing network) (S1 Supporting Information).

### Statistical analyses

We fit both linear probability models and logistic regression models to estimate the influence of these peer adoption rates and other factors, on the individual adoption outcome for each physician. Comparing the estimated outcomes obtained from linear and logistic models, we found that the mean and median differences of the estimated values were ≤0.1% and the first and third quartiles of the difference were within 3%. Accordingly, and to allow us to easily implement instrumental variables methods, we used linear probability models specified as follows:

$$\begin{array}{c} y_{i}=\beta_{0}+x_{i}^{'}\beta_{1}+\gamma_{P}{\overset{-}{y}}_{Pi}+\gamma_{G}{\overset{-}{y}}_{Gi}+\gamma_{H}{\overset{-}{y}}_{Hi}+\gamma_{T}{\overset{-}{y}}_{Ti}+\lambda_{P}{\overset{-}{c}}_{Pi}+\lambda_{G}{\overset{-}{c}}_{Gi}+\lambda_{H}{\overset{-}{c}}_{Hi}+\lambda_{T}{\overset{-}{c}}_{Ti}\\ +\delta_{P}{\overset{-}{v}}_{Pi}+\delta_{G}{\overset{-}{v}}_{Gi}+\delta_{H}{\overset{-}{v}}_{Hi}+\delta_{T}{\overset{-}{v}}_{Ti}+\varepsilon_{i} \end{array}$$

where *y*_*i*_ is the binary indicator of adoption. Covariates included: characteristics of the individual physician (*x*_*i*_) such as demographics, training, specialty, prescribing volume, and location; the age distribution and payer mix of the physician’s patients filling prescriptions (also included in *x*_*i*_); and the four variables for peer adoption rates in each network (${\bar{y}}_{Pi}$ to ${\bar{y}}_{Ti}$)–the coefficients on these variables being key estimates of interest (S1 Supporting Information for additional details). We checked the overlap in connections among these 4 networks and determined that it was low (<20%) allowing us to estimate the effect of all 4 simultaneously in the same model.

The estimation of peer effects with observational data has many challenges.[44–46] The most basic problem is that each physician influences her peers just as they influence her (simultaneity). In addition, physicians may choose peers who are similar to them (homophily), and there may be other, unobserved factors that are common among groups of peers. To address these sources of bias we use an instrumental variables approach to estimation that is common in the econometric literature on peer effects.[47] A set of exogenous characteristics of peers, such as their sex, age, and location, serve as “instruments” which predict peer adoption rates in the absence of confounding factors. Estimation proceeds via two-stage least squares. In the first stage, the means of exogenous peer characteristics were used to predict the adoption rates in each peer network (S1 Supporting Information). In the second stage, the predicted adoption rates were then used in place of the observed adoption rates to estimate our main model.

This approach yields consistent estimates as long as peer characteristics meet two criteria. First, they must have no *direct* influence on an individual’s adoption outcome and must not be correlated with unobserved confounders. For example, the proportion of peers who are female should not directly affect whether a physician adopts the drug, net of the adoption rate among those peers. By contrast, we believe the proportion of peers who are relevant specialists or high-volume prescribers may have a direct influence on physician adoption. For example, a primary care physician may be less likely to adopt a new drug if he can easily refer complex patients to specialists in the same hospital. Hence, neither the specialty mix nor the prescribing volume of peers are used as instruments. Instead, we include eight variables for the proportions of peers in each network who are relevant specialists (${\bar{c}}_{Pi}$ to ${\bar{c}}_{Ti}$) or are high-volume prescribers (${\bar{v}}_{Pi}$ to ${\bar{v}}_{Ti}$) in the main model. The assumption that the other peer characteristics are valid instruments can be partially assessed,[48] and this assessment is presented in detail in the S1 Supporting Information. Overall the results indicate that our instruments are not correlated with unobserved confounders: the null hypothesis of no confounding is not rejected for two out of the three drugs (p = 0.213 for sitagliptin, p = 0.500 for sitagliptin). The diagnostic testing for dabigatran suggests that unmeasured confounding could be an issue (p = 0.031), but given that a rejection of the null can also occur as a consequence of functional misspecification (e.g., variable definitions, presence or absence of interactions, etc.), and because there is no known reason why this drug would be different from the others, we maintain dabigatran in the present analysis.

Second, the means of peer characteristics must be sufficiently predictive of the peer adoption rates. We assessed this with two sets of tests for weak instruments commonly used in the literature.[49] For all three drugs the peer characteristics were sufficiently predictive in the patient-sharing and hospital networks, with first-stage F-statistics above 10, but not in the medical group and training networks. We then tested whether the instruments were sufficiently predictive for the patient-sharing and hospital networks jointly, using the minimum eigenvalue statistic, which confirmed that the instruments have sufficient power in these two networks (S8 Table). As a consequence of this assessment, we consider the estimated peer effects in the patient-sharing and hospital networks to be statistically reliable, but not those in the medical group or training networks.

After quantifying the magnitude of peer influence in each network, we used our estimates to compute an aggregate “social multiplier” on adoption[50] (S1 Supporting Information). Conceptually, the social multiplier represents the number of other physicians expected to adopt a new drug following adoption by a given individual physician. This reflects both the direct influence a physician has on her own peers as well as her indirect influences on others throughout the network. The formula for the social multiplier in the patient-sharing network, our focal network, is as follows:

$$\gamma_{P}\sum_{j\neq i}w_{ji}+\gamma_{P}^{2}\sum_{j\neq i}\sum_{k\neq j}w_{ji}w_{kj}+\gamma_{P}^{3}\sum_{j\neq i}\sum_{k\neq j}\sum_{l\neq k}w_{ji}w_{kj}w_{lk}+\cdots$$

where *w*_*ji*_ is the weight on the link between physicians *j* and *i* based on the number of shared patients, and *γ*_*P*_ is the coefficient on the peer adoption rate from the linear probability model of adoption. The social multiplier is an eigenvector centrality equivalent to a type of Bonacich power centrality[51] in a weighted, directed graph, where *γ*_*P*_ serves as the attenuation parameter. We calculated the social multiplier for each physician in the network, and then used this to conduct a simulation using the patient-sharing network and adoption of dabigatran to illustrate. Imagine a health system wishing to achieve rapid diffusion of a new technology seen as cost-effective for treating a condition. In order to target scarce resources efficiently that system could target interventions to physicians seeing a high volume of the relevant patient population. Alternatively, the payer could target interventions to physicians with many peer connections observed in administrative claims data. To illustrate the value of targeting physician interventions based on social network vs. other physician characteristics, we present the average multiplier (i.e., number of other physicians who would adopt a drug) by physician prescribing volume, and a simple measure of network centrality: the number of direct peer connections a physician has (through patient-sharing), also known as degree.[52]

We used SAS 9.4 (Cary, NC) for data management and variable construction, the network analysis library Igraph in python[36] to construct the peer networks, STATA V14 to estimate the instrumental variable models, and R x64 3.3.2 to compute the multiplier described above. This study was approved by the University of Pittsburgh Institutional Review Board.

## Results

### Descriptive characteristics of physicians

We examined adoption of new drugs among 7,785 physicians prescribing anticoagulants, 8,257 prescribing antidiabetic medications, and 9,974 prescribing antihypertensives. Physicians were, on average, 49–50 years old and roughly one-quarter were female (Table 1). Most (61–72%) physicians prescribing these medications were in primary care specialties (e.g., internal medicine). The medical sub-specialties of interest–cardiology, nephrology, and endocrinology–made up 3–14% of physicians depending on the cohort. Most physicians were affiliated with at least one medical group (69–75%) and hospital (90%). The proportion of physicians adopting the new drugs varied; one-quarter (25.2%) of anticoagulant prescribers adopted dabigatran, a similar fraction of antidiabetic prescribers adopted sitagliptin (24.5%), but fewer antihypertensive prescribers adopted aliskiren (8.3%) (Table 1). Several characteristics differed significantly in bivariate analyses between adopters and non-adopters including the percent who were specialists and had high prescribing volumes (S3–S5 Tables).

**Table 1:** Characteristics of prescriber cohorts for each class of new drug.

	Anticoagulants	Antidiabetics	Antihypertensives
**N**	7,785	8,257	9,974
**Mean age (years) ± SD**	50.6 ± 10.5^a^	48.7 ± 10.0^b^	49.4 ± 10.1^b^
**Years since medical school graduation**			
**<10**	827 (10.6)	1044 (12.6)	1244 (12.5)
**10–19**	2043 (26.2)	2362 (28.6)	2732 (27.4)
**20–29**	2576 (33.1)	2943 (35.6)	3581 (35.9)
**30+**	2339 (30.0)	1908 (23.1)	2417 (24.2)
**% female**	24.8%	27.0%	24.8%
**Primary Specialty**			
**Cardiology**	1042 (13.4)	NA^c^	1106 (11.1)
**Endocrinology**	NA^c^	274 (3.3)	NA^c^
**Nephrology**	NA^c^	NA^c^	311 (3.1)
**PCP**	5579 (71.7)	5748 (69.6)	5959 (60.8)
**Other Physicians**	1164 (15.0)	2235 (27.1)	2598 (26.0)
**Has ≥1 medical group affiliation (%)**	5854 (75.2)	5862 (71.0)	6848 (68.7)
**Has ≥ 1 hospital affiliation (%)**	7032 (90.3)	7446 (90.2)	8976 (90.0)
**Prescribing volume**^d^			
**Mean (SD)**	143.0 (99.3)	253.6 (217.2)	105.7 (71.3)
**Median**	149.2	178.4	116.3
**Payer mix**			
**Cash**	3.6% ± 5.6%	4.0% ± 7.4%	4.9% ± 8.1%
**Commercial**	50.0% ± 21.2%	57.9% ± 23.8%	60.4% ± 21.4%
**Medicaid fee-for-service**	5.5% ± 12.1%	8.5% ± 15.1%	6.7% ± 12.4%
**Medicare**	41.0% ± 19.7%	29.6% ± 19.9%	28.0% ± 17.3%
**Patient age mix**			
**0–64**	37.3% ± 23.2%	54.9% ± 24.7%	55.0% ± 22.3%
**65–74**	22.5% ± 15.5%	22.3% ± 16.6%	20.2% ± 13.7%
**75–84**	26.2% ± 16.9%	16.7% ± 15.7%	16.8% ± 13.4%
**85+**	14.1% ± 13.8%	5.9% ± 9.5%	8.0% ± 9.5%
**Location**			
**Rural**	910 (11.7)	914 (11.1)	1020 (10.2)
**Metropolitan**	6875 (88.3)	7343 (88.9)	8954 (89.8)
**Medical school location**			
**US**	6090 (78.2)	6489 (78.6)	7827 (78.5)
**Foreign**	1695 (21.8)	1768 (21.4)	2147 (21.5)
**Medical school ranking**			
**Top 20**	769 (9.9)	880 (10.7)	1127 (11.3)
**Non-Top 20**	7016 (90.1)	7377 (89.3)	8847 (88.7)
**Hospital referral region**			
**Allentown**	690 (8.9)	692 (8.4)	837 (8.4)
**Altoona**	153 (2.0)	151 (1.8)	170 (1.7)
**Danville**	309 (4.0)	285 (3.5)	340 (3.4)
**Erie**	339 (4.4)	339 (4.1)	384 (3.9)
**Harrisburg**	612 (7.9)	596 (7.2)	723 (7.3)
**Johnstown**	131 (1.7)	124 (1.5)	140 (1.4)
**Lancaster**	375 (4.8)	373 (4.5)	404 (4.1)
**Philadelphia**	2409 (30.9)	2808 (34.0)	3549 (35.6)
**Pittsburgh**	1657 (21.3)	1785 (21.6)	2179 (21.9)
**Reading**	337 (4.3)	337 (4.1)	383 (3.8)
**Sayre**	72 (0.9)	78 (0.9)	87 (0.9)
**Scranton**	193 (2.5)	192 (2.3)	228 (2.3)
**Wilkes-Barre**	166 (2.1)	158 (1.9)	190 (1.9)
**York**	233 (3.0)	235 (2.9)	253 (2.5)
**Non-PA HRR**	109 (1.4)	104 (1.3)	107 (1.1)
**Percent adopting new drug in first 15 months**	25.2%	24.5%	8.3%

Data sources: QuintilesIMS, HCOS; XPonent; AMA Masterfile

^a^ age of physician in 2010,

^b^ age of physician in 2007

^c^ not a sub-specialty of interest for this particular class of drugs so included in “other physicians”

^d^ Prescribing volume is at the class-level and is measured as total prescriptions dispensed during the period when adoption was measured that were written by each physician.

In each cohort, the patient-sharing network had the greatest number of peers, ranging from 200–344 depending on the cohort, with a small share of providers (2–6%) having no peers in the patient-sharing network **(**S7 Table). Physicians in each cohort shared at least one hospital affiliation with an average of 166–204 peers. Physicians had fewer peers in the medical group (9–12) and training networks (39–46).

### Unadjusted association between peer adoption and own adoption

**Fig 2:**
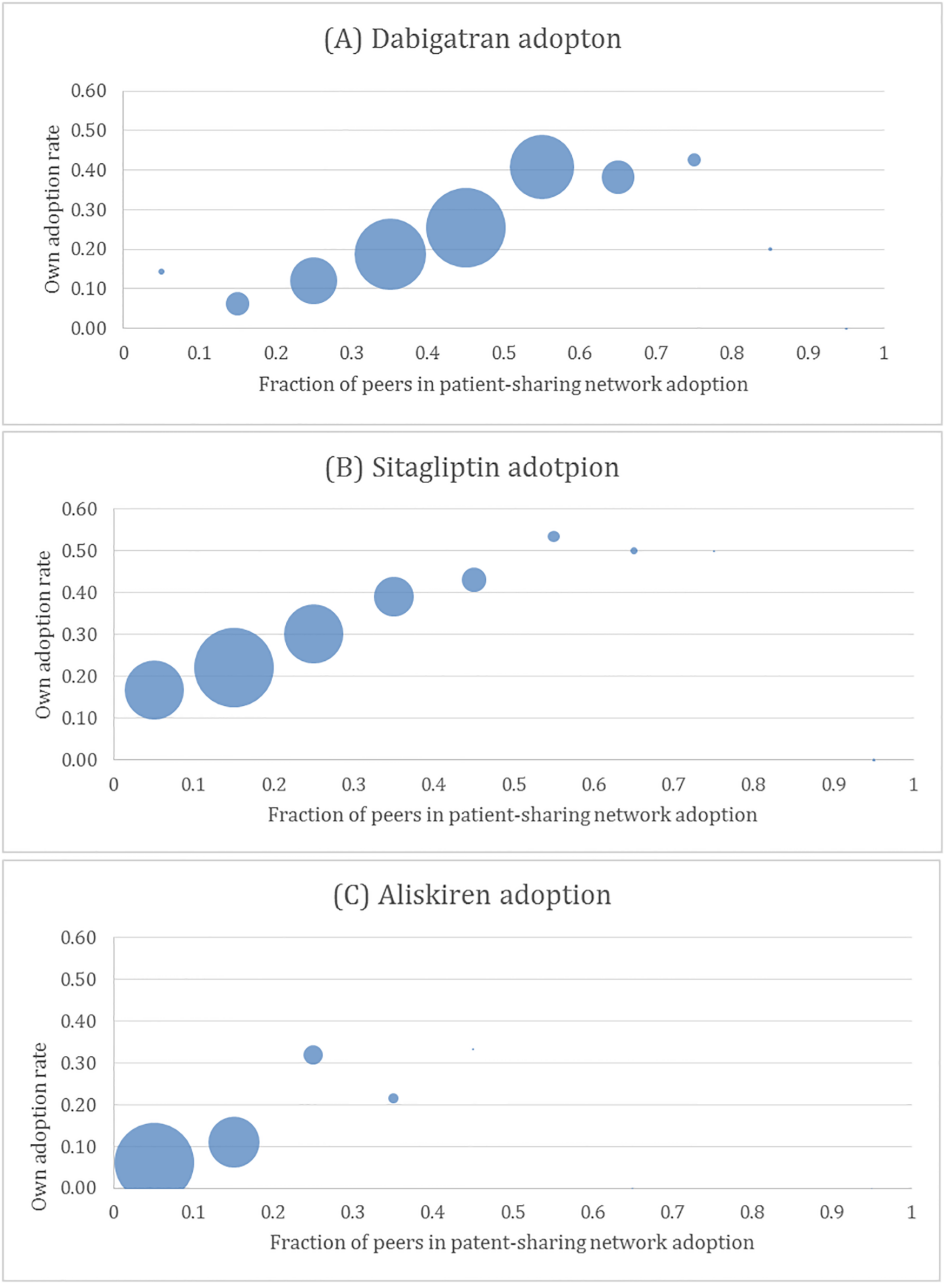
a-c: Unadjusted relationship between physicians’ own adoption rate and that of their peers in the patient-sharing network. The graphs show the average physicians’ own adoption rate for a given fraction of peers in the patient-sharing network adopting the drug. The size of the bubble corresponds to the number of physicians with a particular peer adoption rate. The graphs show a positive association between the fraction of peers adopting the new drug and the physicians’ own likelihood of adopting. For example, with dabigatran, physicians in networks in which the fraction of peers adopting is 0.65 have a 0.38 probability of adopting compared to physicians whose peers’ adoption rate is 0.35 who have only a 0.19 probability of adopting.

In unadjusted analyses, a physician’s likelihood of adopting a drug was strongly associated with the likelihood that her peers had also adopted the drug. For example, a physician for whom 2/3 of her peers had adopted dabigatran was twice as likely to adopt the drug compared to a physician for whom one-third of her peers had adopted the same drug (Fig 2a).

### Adjusted estimates of peer effects on adoption

**Fig 3:**
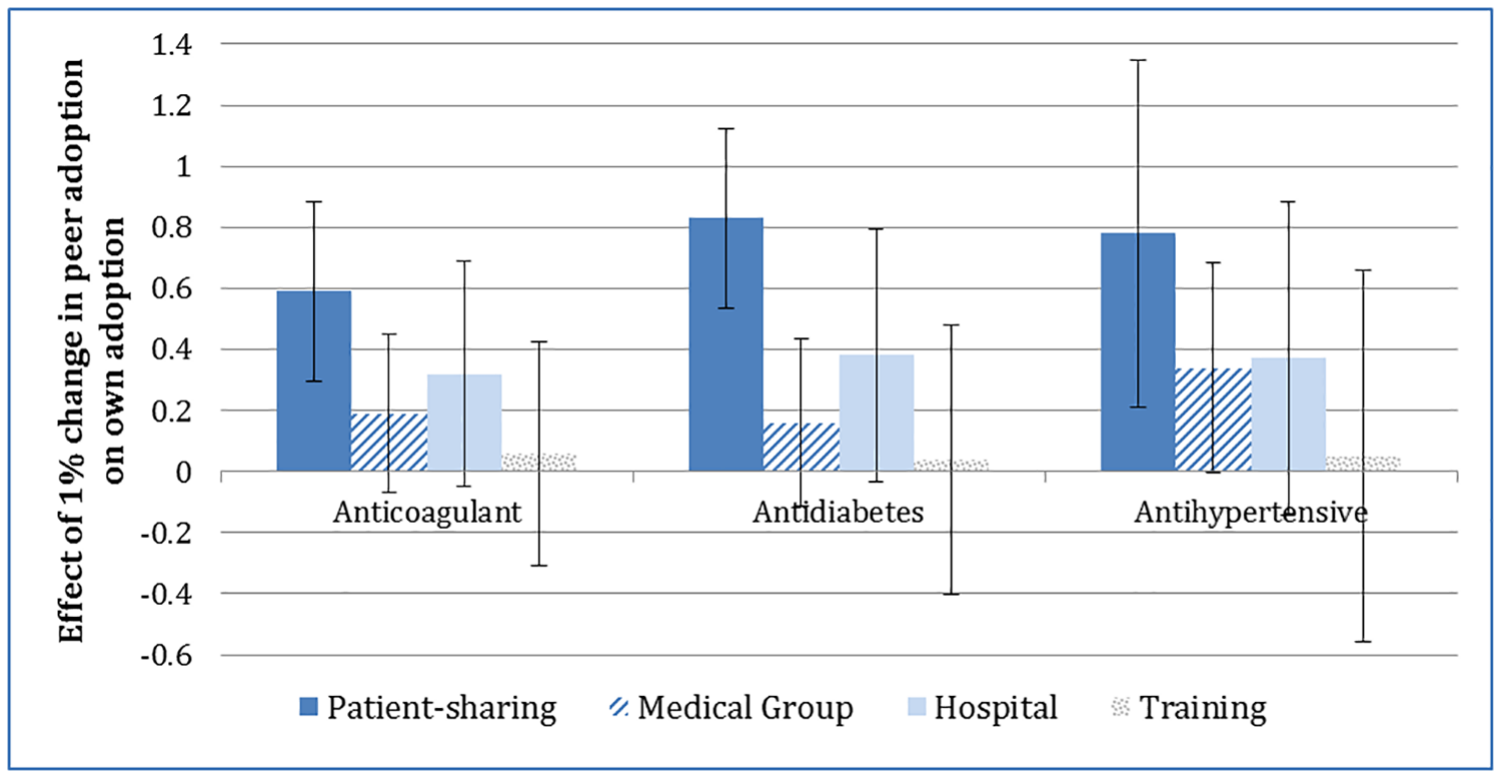
Estimated peer effects on drug adoption with 95% confidence intervals. Estimates show the effect of a 1% absolute change in the adoption rate of a physician’s peers on the likelihood of a physician’s decision to adopt the new drug. For example, a 1% increase in patient-sharing network peer adoption of dabigatran corresponded to a 0.59% increase in the probability of own adoption. Estimates are from a two-stage least squares regression model including physician-level characteristics: sex, medical school graduation year, US vs. non-US medical school, Top 20 U.S. medical school vs. not, geographic indicators (hospital referral region and metropolitan vs. non-metropolitan, prescription share paid for by Medicare, Medicaid fee-for-service, and cash, and age of patients filling prescriptions (see S10 Table for full model estimates). The means of peer characteristics serve as instruments for peer adoption rates. Included in the second stage were: physician specialty (primary care physician, relevant sub-specialty; e.g., cardiologist, endocrinologist, nephrologist vs. other), proportion of peers in each network who are in each specialty group, an indicator for whether the physician was a high-volume prescriber (> = median), the proportion of peers in each network who were high-volume prescribers, and an indicator for physicians who do not have peers in a particular type of network in the cohort. Peer effects estimates in the medical group and training networks are not reliable due to poor predictive power of the instruments.

In the fully-adjusted instrumental variables analysis, peer effects on adoption were largest in the patient-sharing network for all three drugs (Fig 3). For example, among anticoagulant prescribers, the coefficient for peer effects in the patient-sharing network was 0.590 (standard error (SE) = 0.150, p<0.001). This implies that for every 10 percentage point increase in the fraction of peers in the patient-sharing network adopting dabigatran, a physician’s own adoption probability increased by 5.90%. The coefficients on peer adoption in the patient-sharing network were of similar magnitude for the other two new medications. A 10 percentage point increase in peer adoption corresponded to a 8.32% (SE = 1.51%, p<0.001) increase in sitagliptin adoption, and a 7.84% increase in aliskiren adoption (SE = 2.93%, p<0.001) (S10 Table displays full model estimates).

After adjusting for other peer effects and covariates, own adoption was positively influenced by peer adoption in the same hospital although the effect was not statistically significant for any of the drugs at p<0.05 (Fig 3). The coefficient for hospital peer adoption was 0.319, SE = 0.188, p = 0.09 for dabigatran; 0.376, SE = 0.211, p = 0.08 for sitagliptin; and 0.367, SE = 0.261, p = 0.16 for aliskiren. Estimates of medical group and training network effects were not reliable for any of the three drugs due to weak instruments.

### Illustration of use of social networks to target interventions

**Fig 4:**
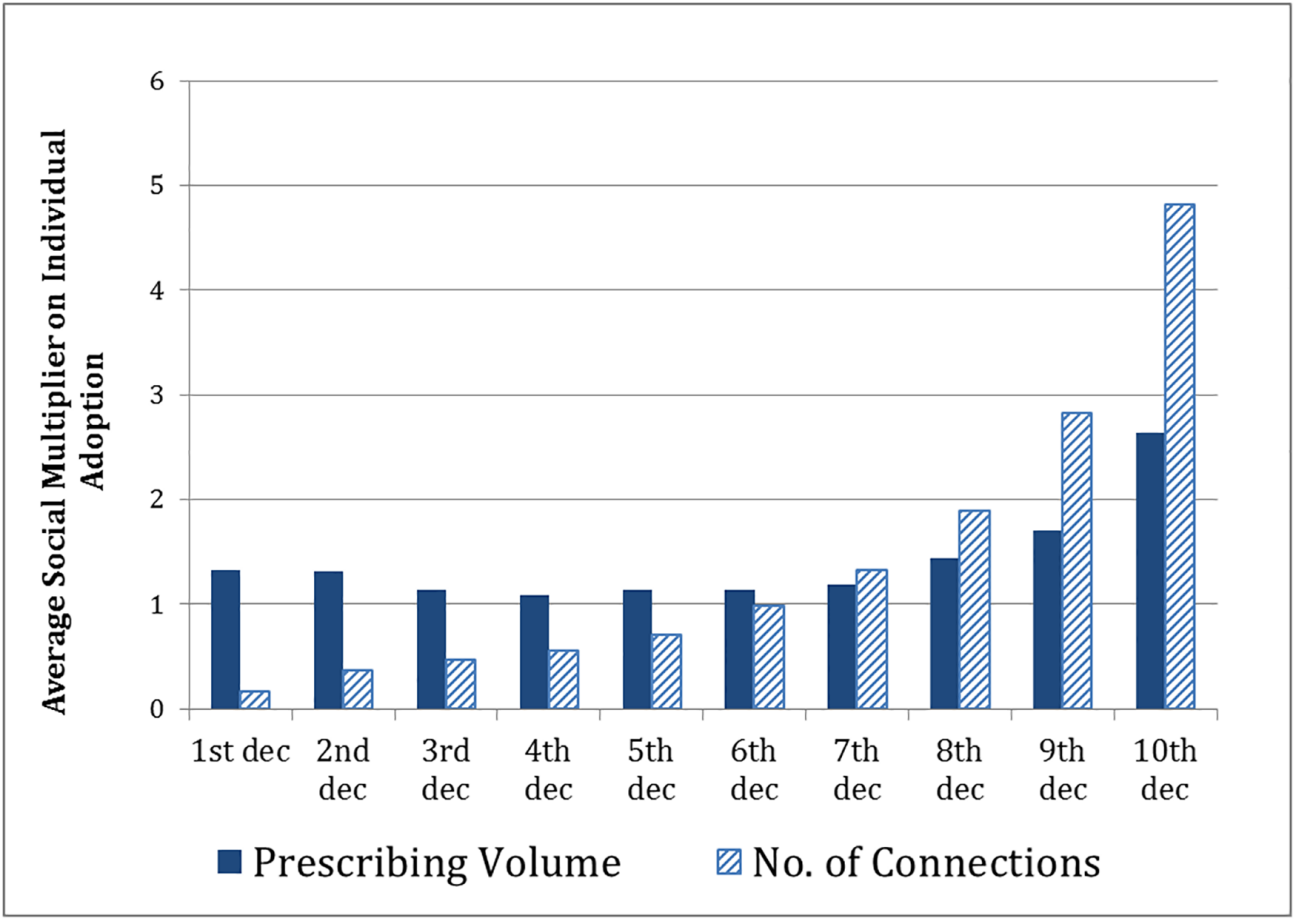
Physicians’ average social multiplier by prescribing volume and number of connections in the patient-sharing network. The social multiplier captures the number of other physicians who might adopt dabigatran following adoption by an individual physician in their peer network. Thus, the figure shows the simulated number of physicians who would adopt dabigatran per physician in each decile of prescribing volume (dark blue bars) and number of connections in the patient-sharing network (striped bars) adopting dabigatran. Physicians in the top decile of patient-sharing who had the most connections with other physicians who adopted dabigatran appear to have a nearly 2-fold stronger influence on adoption by other physicians compared to physicians in the top decile of prescribing volume (4.81 vs. 2.64).

Fig 4 shows the social multipliers using the adoption of dabigatran in the patient-sharing network to illustrate the potential for using social network information to target interventions. The figure presents the average multiplier by decile of peer connections (i.e., number of physicians with whom a physician shares patients) and by decile of prescribing volume and shows the value of targeting interventions using peer network information. Adoption by physicians in the top decile of connections is projected to induce 28 times as many other physicians to adopt compared with physicians in the bottom decile (4.81 vs. 0.16 physicians adopting). By contrast, targeting physicians in the top volume decile is projected to induce only twice as many adoptions as targeting physicians in the bottom decile (2.64 vs. 1.33 physicians adopting), and little more than half as many as targeting the top decile of connections (2.64 vs. 4.81).

## Discussion

We find that physician adoption of new drugs is heavily influenced by the extent to which their peers have adopted those new drugs. These effects were particularly large for peers with whom physicians share patients. Our study points to a potential mechanism underlying the tremendous variation in US medical care and to the importance of viewing physicians as part of a larger social system.

We sought to measure and test the effects of a rich set of peer influences derived from the institutional affiliations that physicians have with medical group practices, hospitals, and health systems. We also examined the influence of peers in the informal networks physicians form as they develop referral relationships, and interact with other physicians in the management of shared patients. Notably, peers in these informal patient-sharing networks had the largest effects on adoption of all three drugs after adjusting for the influence of peers in the same medical group and hospital setting. The patient-sharing network may exert the most influence on physician adoption because it captures more active connections over which physicians exercise the most control. A physician likely has more discretion, for example, over whom he refers his patients to than he does over the physicians with admitting privileges to the same hospital. This element of choice in the patient-sharing network raises the possibility that our estimates may be influenced in part by homophily[53, 54] although our use of instrumental variables reduces this as a concern. A key advantage of using patient-sharing information to measure peer networks is the routine availability of claims data to payers, the stakeholders best-positioned to invest in large-scale interventions to improve the quality of care.

The magnitude of the patient-sharing peer effects on physician adoption was broadly consistent across three drugs introduced over a 4-year time period that were prescribed to different patient populations, and by physicians with primary care and medical sub-specialty training. We studied the introduction of three newly-approved drugs that were first-in-class, each with a novel mechanism of action. All three medications have a place in the treatment armamentarium alongside older, existing therapies but varied with respect to whether they are considered first- or second-line treatments. That the magnitude of peer influence on adoption was comparable for all three of these drugs adds to the generalizability of our findings.

Meltzer and colleagues describe an approach to using social network analysis to form quality improvement teams to maximize the reach of interventions.[11] Similarly, our social multiplier exercise illustrates that targeting interventions to physicians who are well-connected in patient-sharing networks may be a more efficient way of improving the diffusion of evidence-based therapies. For example, health systems routinely adopt intensive educational interventions such as academic detailing—face-to-face educational programs borrowing principles from pharmaceutical promotional efforts. These educational interventions may reduce over-prescribing of ineffective medications, or increase use of highly effective agents.[7] Because academic detailing visits are meant to be in-depth and frequent[55] they are difficult to deliver on a large scale. However, systematic reviews suggest that academic detailing is seldom targeted.[56] Our study relates to an extensive literature on speeding diffusion of innovations in healthcare and other sectors by targeting ‘key opinion leaders’ who by virtue of their technical competence, credibility, and/or social acceptability are seen as influential with their peers.[57–60] What our study adds to this body of work is information on the potential for using patient-sharing based measures of network centrality to target influential physicians at a larger scale than a single health care setting or community.[61] We focus on the most basic centrality measure, e.g., degree centrality, because it is simple to construct facilitating its widespread use and does not require complete observation of the network. Other centrality measures such as eigenvector centrality, Bonacich centrality, PageRank centrality, density centrality have been used to characterize other technological diffusion processes[51, 62–64] and may be useful in this context.

Our study improves on prior work by including a larger physician sample, examining the diffusion of multiple new drugs, drawing on several information sources to form 4 types of physician networks, and using an instrumental variables approach to overcome some challenges in estimating network effects. Nevertheless, we note some limitations. First, although Pennsylvania resembles national averages on most measures of healthcare utilization,[25] our findings are limited to a single state and estimates of peer effects on physician adoption may not generalize to other geographic areas with different physician network structures[38] or adoption patterns. Second, while our study of adoption of three new prescription drugs improves on prior studies of uptake of a single technology, our estimates of peer effects may not generalize to all medical technologies. Third, while we adjusted for several physician characteristics associated with adoption we lack information on important sources of influence on physician behavior, namely patient clinical characteristics, pharmaceutical company promotion, and payer formularies. Only having access to information on the age and source of payment for a physician’s patients who filled their prescriptions we are unable to adjust for differences in adoption due to patient health state. We note that all three of the new drugs were first-in-class and likely to be heavily promoted by manufacturers; however, our study predates availability of physician-level data on exposure to industry promotion. We were not able to adjust for differences in formulary coverage of the new drugs for a physician’s patient panel. Fourth, as with any study that uses instrumental variables, our estimates capture the effects of peer adoption rates that were driven by the peer mean characteristics we use as instruments, not by other sources of variation, which potentially limits their generalizability.

Using multiple data sources to measure the rich set of peer relationships formed by physicians we find that peers can exert significant influence over physician technology adoption decisions. Our study shows the potential for using information on physician social networks routinely available to health systems to improve the targeting of interventions to speed the diffusion of evidence-based health care technologies.

## Supporting information

S1 Fig Sample size flow chart for anticoagulant prescribers. **Sources**: QuintilesIMS’s XPonent, QuintilesIMS’s HCOS, and AMA Masterfile **Abbreviations**: AC: Anticoagulant; PA: Pennsylvania. **Notes**: Dabigratran was introduced in October 2010 and our XPonent data end in December 2011. We included physicians with at least some minimal anticoagulant prescribing, although not necessarily the drug of interest, and an AMA Masterfile record and HCOS record for the maximum duration given data availability (15 months post-market introduction).
(DOCX)

S2 Fig Sample size flow chart for antidiabetic prescribers. **Sources**: QuintilesIMS ‘s XPonent, QuintilesIMS’s HCOS, and AMA Masterfile **Abbreviations**: AD: Antidiabetic; PA: Pennsylvania. **Notes**: Sitagliptin was introduced in October 2006 a few months before our data was available. We included physicians with at least some minimal antidiabetic prescribing, although not necessarily the drug of interest, and an AMA Masterfile and HCOS record from months 3 to 15 following sitagliptin’s introduction, the period during which adoption is measured.
(DOCX)

S3 Fig Sample size flow chart for antihypertensive prescribers. **Sources**: QuintilesIMS’s XPonent, QuintilesIMS’s HCOS, and AMA Masterfile **Abbreviations**: AH: Antihypertensive; PA: Pennsylvania. **Notes**: Aliskiren was introduced in March 2007. To provide a comparable period over which adoption is measured for all classes we included physicians with at least some minimal antihypertensive prescribing, although not necessarily the drug of interest, and an AMA Masterfile record and HCOS record for 15 months following aliskiren’s introduction.
(DOCX)

S1 Table A) Oral Anti-coagulant products; B) Antihypertensive products in ACEI, ARB, and RAAS classes; C) Oral anti-diabetic medications. (DOCX)

S2 Table Distribution of number of prescriptions for the new drugs among physicians adopting them. (DOCX)

S3 Table Characteristics of anticoagulant prescriber cohort and comparisons by adoption vs. non-adoption of new drug (dabigatran). Data sources: QuintilesIMS, HCOS; XPonent; AMA Masterfile ^1^ age of physician in 2010.
(DOCX)

S4 Table Characteristics of anti-diabetes medication prescriber cohort and comparisons by adoption vs. non-adoption of new drug (sitagliptin). Data sources: QuintilesIMS, HCOS; XPonent; AMA Masterfile * Age in 2007.
(DOCX)

S5 Table Characteristics of antihypertensive prescriber cohort and comparisons by adoption vs. non-adoption of new drug (aliskiren). Data sources: QuintilesIMS, HCOS; XPonent; AMA Masterfile. *Age in 2007.
(DOCX)

S6 Table Sample size of physicians in each prescribing cohort with Medicare and/or Medicaid claims during adoption measurement period. Sources: Medicare data were obtained from CMS. Medicaid data were obtained from the Pennsylvania Department of Human Services. Notes: In the anticoagulant cohort, there are 7,785 physicians meeting inclusion criteria of whom 7,522 (96.6%) had Medicare claims and 6,680 (85.8%) had Medicaid claims. We included claims submitted by those physicians to Medicare and Medicaid with dates of service between 10/1/2010 and 12/31/2011 to match the period over which we measured adoption of dabigatran. In the antidiabetic cohort, 8,257 physicians met inclusion criteria. We included claims from Medicare and Medicaid submitted by those physicians with dates of service between 1/1/2007 and 1/31/2008 (the measurement period for adoption of sitagliptin). There are 9,974 physicians meeting inclusion criteria for the antihypertensive prescriber cohort. We included claims from Medicare or Medicaid with dates of service between 3/1/2007 and 5/31/2008, the measurement period for adoption of aliskiren.
(DOCX)

S7 Table Number of physician peers to which physicians are connected in patient-sharing, medical group, hospital and training networks. Data sources: QuintilesIMS, HCOS; XPonent; AMA Masterfile 1 Column displays the number of physicians who are not connected to any peers in a particular network. For example, physicians may lack peers in the patient-sharing network because they do not see patients with Medicaid and Medicare patients or because they did not share any patients with that source of coverage with other physicians in the prescribing cohort.
(DOCX)

S8 Table Assessments of instrument exogeneity and relevance. (DOCX)

S9 Table Peer characteristics used as instruments in each network. Data sources: QuintilesIMS, HCOS; XPonent; AMA Masterfile *In the analytical dataset, the unit is physician. For categorical variables, we obtain the proportion of each type (e.g., proportion of peers who are female) for each physician. For the continuous variables, we use the average percentage of pay type (e.g., Medicare) of all peers for each physician. Table shows the proportion of physicians in each network with that characteristic.
(DOCX)

S10 Table Estimates of peer effects and covariates on adoption of new drugs from instrumental variables regression. ***Primary analysis showing all covariates*. Data sources**: QuintilesIMS, HCOS; XPonent; AMA Masterfile Notes: Robust standard errors in parentheses *** p<0.001, ** p<0.01, * p<0.05 ^a^high-volume = total prescribing volume in the category above the median.
(DOCX)

S11 Table Estimates of peer effects on adoption of new drugs from linear model without instrumental variables. **Data sources**: QuintilesIMS, HCOS; XPonent; AMA Masterfile Notes: Robust standard errors in parentheses *** p<0.001, ** p<0.01, * p<0.05.
(DOCX)

S12 Table Estimates of peer effects and covariates on adoption of new drugs. ***Alternate specification of own and peer adoption (using> = 1 and 15)***. Data sources: QuintilesIMS, HCOS; XPonent; AMA Masterfile Notes: Robust standard errors in parentheses *** p<0.001, ** p<0.01, * p<0.05.
(DOCX)

S13 Table Distribution of aggregate social multipliers on individual adoption by drug and network. (DOCX)

S1 Supporting Information (DOCX)
