# Supplementary figures and images for: Influence of peer networks on physician adoption of new drugs
Source: PLoS One. 2018 Oct 1;13(10):e0204826. doi: 10.1371/journal.pone.0204826 (PMC6166964; doi:10.1371/journal.pone.0204826)

**S1 Figure. Sample size flow chart for anticoagulant prescribers**

**
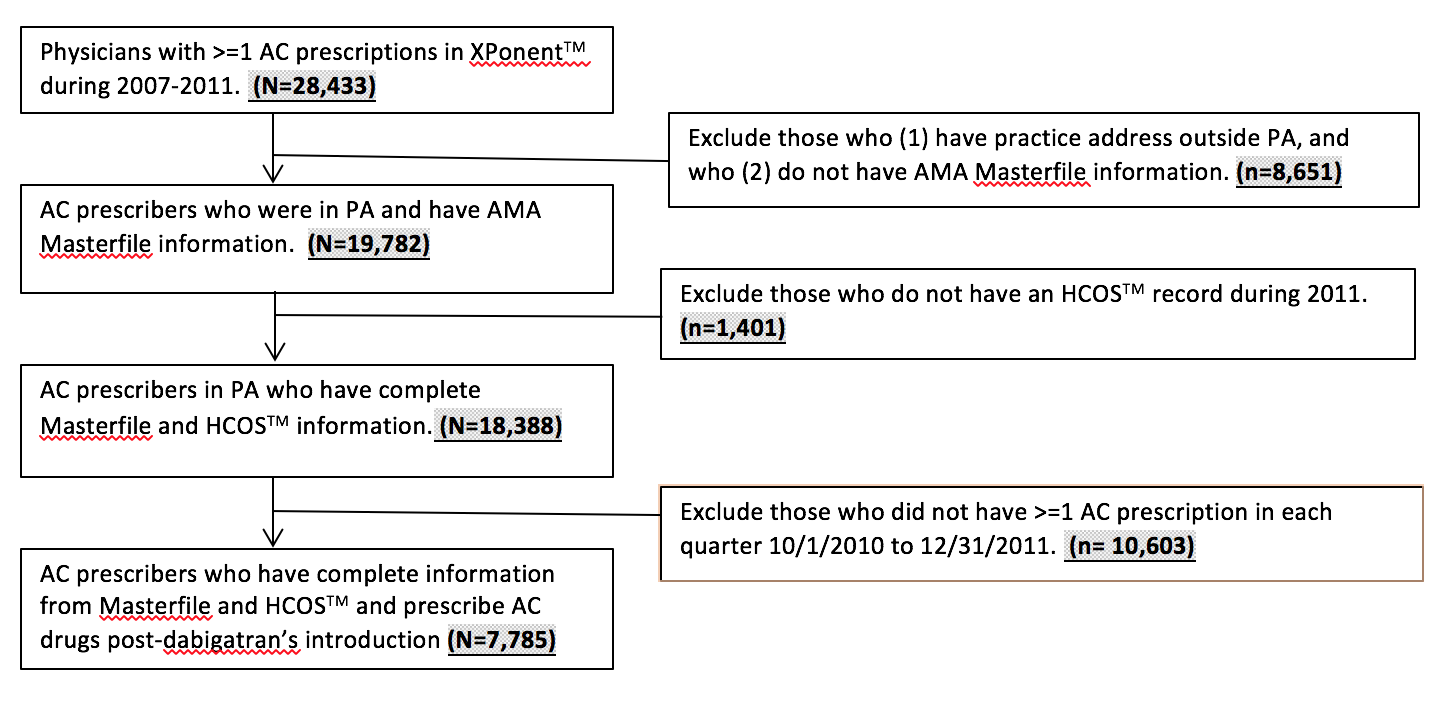
**

Supplement: S1 Fig — Sources: QuintilesIMS’s XPonent, QuintilesIMS’s HCOS, and AMA Masterfile Abbreviations: AC: Anticoagulant; PA: Pennsylvania. Notes: Dabigratran was introduced in October 2010 and our XPonent data end in December 2011. We included physicians with at least some minimal anticoagulant prescribing, although not necessarily the drug of interest, and an AMA Masterfile record and HCOS record for the maximum duration given data availability (15 months post-market introduction). (DOCX) [file pone.0204826.s001.docx]

**S2 Figure. Sample size flow chart for antidiabetic prescribers**

***
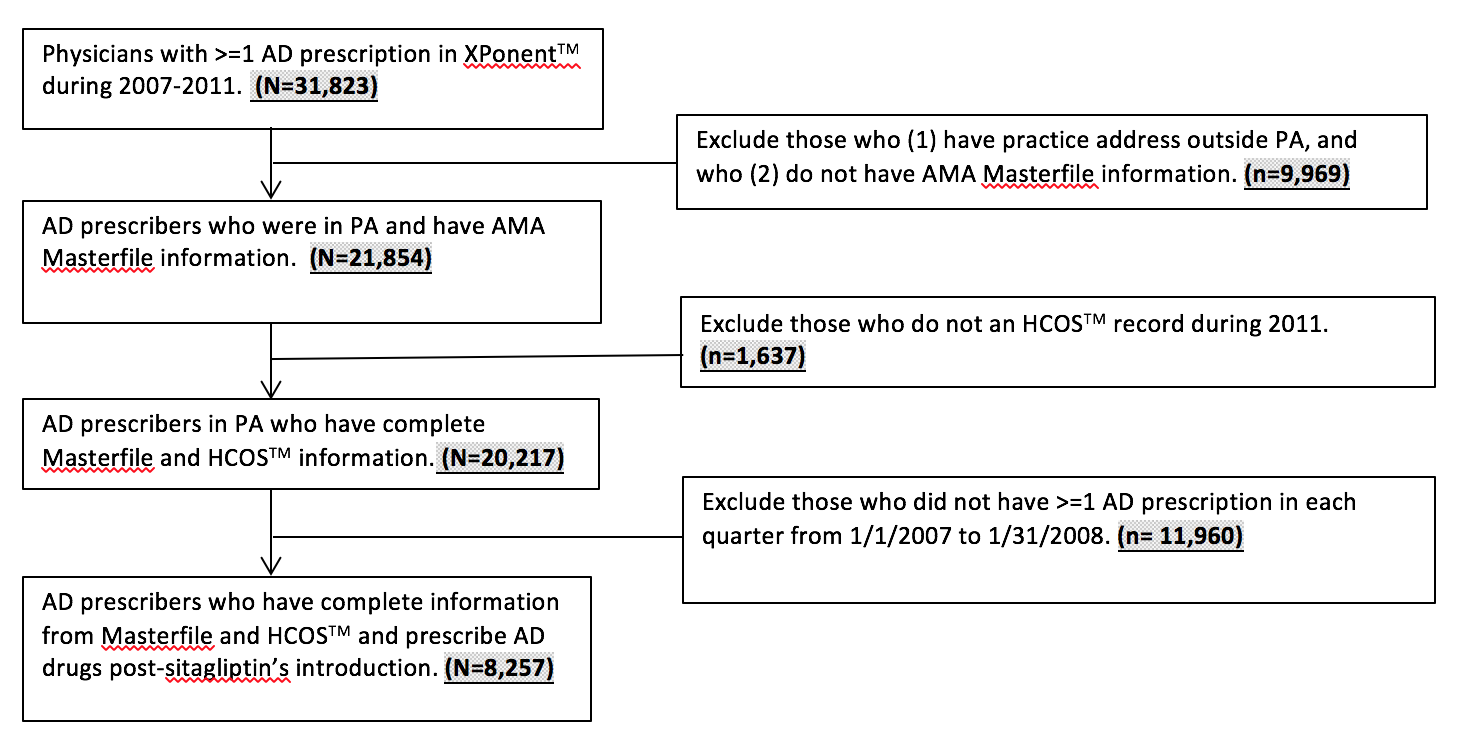
***

Supplement: S2 Fig — Sources: QuintilesIMS ‘s XPonent, QuintilesIMS’s HCOS, and AMA Masterfile Abbreviations: AD: Antidiabetic; PA: Pennsylvania. Notes: Sitagliptin was introduced in October 2006 a few months before our data was available. We included physicians with at least some minimal antidiabetic prescribing, although not necessarily the drug of interest, and an AMA Masterfile and HCOS record from months 3 to 15 following sitagliptin’s introduction, the period during which adoption is measured. (DOCX) [file pone.0204826.s002.docx]

**S3 Figure. Sample Size Flow Chart for antihypertensive prescribers**


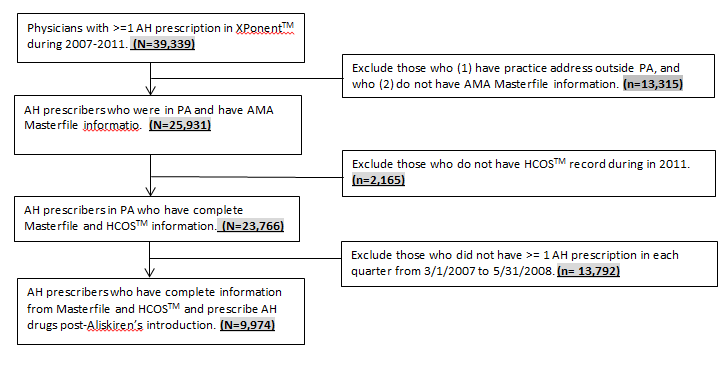

Supplement: S3 Fig — Sources: QuintilesIMS’s XPonent, QuintilesIMS’s HCOS, and AMA Masterfile Abbreviations: AH: Antihypertensive; PA: Pennsylvania. Notes: Aliskiren was introduced in March 2007. To provide a comparable period over which adoption is measured for all classes we included physicians with at least some minimal antihypertensive prescribing, although not necessarily the drug of interest, and an AMA Masterfile record and HCOS record for 15 months following aliskiren’s introduction. (DOCX) [file pone.0204826.s003.docx]
