# Supplementary material for: Influence of peer networks on physician adoption of new drugs
Source: PLoS One. 2018 Oct 1;13(10):e0204826. doi: 10.1371/journal.pone.0204826 (PMC6166964; doi:10.1371/journal.pone.0204826)
Supplement: S1 Table — (DOCX) [file pone.0204826.s004.docx]

**S1a Table: Oral Anti-coagulant products**

| No. | Drug name in Xponent | Tcgpi Name |
| --- | --- | --- |
| 1 | COUMADIN | Warfarin |
| 2 | JANTOVEN | Warfarin |
| 3 | WARFARIN SOD | Warfarin |
| 4 | PRADAXA | Dabigatran |
| 5 | XARELTO | Rivaroxaban |

**S1b Table: Antihypertensive products in ACEI, ARB, and RAAS classes**

| No. | Drug name in Xponent | Tcgpi Name |
| --- | --- | --- |
| 1 | BENAZEPRIL HCL | Benazepril |
| 2 | LOTENSIN | Benazepril |
| 3 | CAPOTEN | Captopril |
| 4 | CAPTOPRIL | Captopril |
| 5 | ENALAPRIL MAL | Enalapril |
| 6 | VASOTEC | Enalapril |
| 7 | ENALAPRILAT | Enalaprilat |
| 8 | FOSINOPRIL SOD | Fosinopril |
| 9 | MONOPRIL | Fosinopril |
| 10 | LISINOPRIL | Lisinopril |
| 11 | PRINIVIL | Lisinopril |
| 12 | ZESTRIL | Lisinopril |
| 13 | MOEXIPRIL HCL | Moexipril |
| 14 | MOEXIPRIL HCL/HCTZ | Moexipril |
| 15 | UNIVASC | Moexipril |
| 16 | ACEON | Perindopril |
| 17 | PERINDOPRIL ERBUMINE | Perindopril |
| 18 | ACCUPRIL | Quinapril |
| 19 | QUINAPRIL HCL | Quinapril |
| 20 | QUINAPRIL HCL/HCTZ | Quinapril |
| 21 | ALTACE | Ramipril |
| 22 | RAMIPRIL | Ramipril |
| 23 | MAVIK | Trandolapril |
| 24 | TRANDOLAPRIL | Trandolapril |
| 25 | EDARBI | Azilsartan |
| 26 | ATACAND | Candesartan |
| 27 | TEVETEN | Eprosartan |
| 28 | AVAPRO | Irbesartan |
| 29 | COZAAR | Losartan |
| 30 | LOSARTAN POT | Losartan |
| 31 | BENICAR | Olmesartan |
| 32 | MICARDIS | Telmisartan |
| 33 | DIOVAN | Valsartan |
| 34 | TEKTURNA | Aliskiren |
| 35 | AMLODIP BES/BENAZ HCL | ACE Inhibitors & Ca Channel Blocker Combination - 2 Ingred |
| 36 | LOTREL | ACE Inhibitors & Ca Channel Blocker Combination - 2 Ingred |
| 37 | TARKA | ACE Inhibitors & Ca Channel Blocker Combination - 2 Ingred |
| 38 | TRANDOL/VERAP HCL | ACE Inhibitors & Ca Channel Blocker Combination - 2 Ingred |
| 39 | ACCURETIC | ACE Inhibitors & Thiazide/Thiazide-Like - Two Ingredient |
| 40 | BENAZEPRIL/HCTZ | ACE Inhibitors & Thiazide/Thiazide-Like - Two Ingredient |
| 41 | CAPTOPRIL/HCTZ | ACE Inhibitors & Thiazide/Thiazide-Like - Two Ingredient |
| 42 | ENALAPRIL MAL/HCTZ | ACE Inhibitors & Thiazide/Thiazide-Like - Two Ingredient |
| 43 | FOSINOPRIL/HCTZ | ACE Inhibitors & Thiazide/Thiazide-Like - Two Ingredient |
| 44 | LISINOPRIL/HCTZ | ACE Inhibitors & Thiazide/Thiazide-Like - Two Ingredient |
| 45 | LOTENSIN HCT | ACE Inhibitors & Thiazide/Thiazide-Like - Two Ingredient |
| 46 | MONOPRIL HCT | ACE Inhibitors & Thiazide/Thiazide-Like - Two Ingredient |
| 47 | PRINZIDE | ACE Inhibitors & Thiazide/Thiazide-Like - Two Ingredient |
| 48 | QUINARETIC | ACE Inhibitors & Thiazide/Thiazide-Like - Two Ingredient |
| 49 | UNIRETIC | ACE Inhibitors & Thiazide/Thiazide-Like - Two Ingredient |
| 50 | VASERETIC | ACE Inhibitors & Thiazide/Thiazide-Like - Two Ingredient |
| 51 | ZESTORETIC | ACE Inhibitors & Thiazide/Thiazide-Like - Two Ingredient |
| 52 | AZOR | Angiotensin II Recept Antag & Ca Chan Block Comb - 2 Ingred |
| 53 | EXFORGE | Angiotensin II Recept Antag & Ca Chan Block Comb - 2 Ingred |
| 54 | TWYNSTA | Angiotensin II Recept Antag & Ca Chan Block Comb - 2 Ingred |
| 55 | ATACAND HCT | Angiotensin II Receptor Antagonists & Thiazides - Two Ingred |
| 56 | AVALIDE | Angiotensin II Receptor Antagonists & Thiazides - Two Ingred |
| 57 | BENICAR HCT | Angiotensin II Receptor Antagonists & Thiazides - Two Ingred |
| 58 | DIOVAN HCT | Angiotensin II Receptor Antagonists & Thiazides - Two Ingred |
| 59 | HYZAAR | Angiotensin II Receptor Antagonists & Thiazides - Two Ingred |
| 60 | LOSARTAN POT/HCTZ | Angiotensin II Receptor Antagonists & Thiazides - Two Ingred |
| 61 | MICARDIS HCT | Angiotensin II Receptor Antagonists & Thiazides - Two Ingred |
| 62 | TEVETEN HCT | Angiotensin II Receptor Antagonists & Thiazides - Two Ingred |
| 63 | EXFORGE HCT | Angiotensin II Receptor Ant-Ca Channel Blocker-Thiazides |
| 64 | TRIBENZOR | Angiotensin II Receptor Ant-Ca Channel Blocker-Thiazides |
| 65 | TEKTURNA HCT | Direct Renin Inhib & Thiazide/Thiazide-Like Comb - 2 Ingred |
| 66 | VALTURNA | Direct Renin Inhib & Angiotensin II Recept Antag - 2 Ingred |
| 67 | TEKAMLO | Direct Renin Inhibitors & Calcium Channel Blocker Comb |
| 68 | AMTURNIDE | Direct Renin Inhibitors-Ca Channel Blocker-Thiazide Comb |
| 69 | CAPOZIDE | ACE Inhibitors & Thiazide/Thiazide-Like - Two Ingredient |
| 70 | LEXXEL | LEXXEL |

**S1c Table: Oral anti-diabetic medications**

| No. | Drug name in Xponent | Tcgpi Name |
| --- | --- | --- |
| 1 | SYMLIN | Pramlintide |
| 2 | SYMLINPEN 120 | Pramlintide |
| 3 | SYMLINPEN 60 | Pramlintide |
| 4 | BYETTA | Exenatide |
| 5 | VICTOZA 2-PAK | Liraglutide |
| 6 | VICTOZA 3-PAK | Liraglutide |
| 7 | CHLORPROPAMIDE | Chlorpropamide |
| 8 | AMARYL | Glimepiride |
| 9 | GLIMEPIRIDE | Glimepiride |
| 10 | GLIPIZIDE | Glipizide |
| 11 | GLIPIZIDE ER | Glipizide |
| 12 | GLUCOTROL | Glipizide |
| 13 | GLUCOTROL XL | Glipizide |
| 14 | DIABETA | Glyburide |
| 15 | GLYBURIDE | Glyburide |
| 16 | GLYBURIDE MICRO | Glyburide |
| 17 | GLYNASE PRESTAB | Glyburide |
| 18 | TOLAZAMIDE | Tolazamide |
| 19 | TOLBUTAMIDE | Tolbutamide |
| 20 | FORTAMET ER | Metformin |
| 21 | GLUCOPHAGE | Metformin |
| 22 | GLUCOPHAGE XR | Metformin |
| 23 | GLUMETZA | Metformin |
| 24 | METFORMIN ER (F) | Metformin |
| 25 | METFORMIN ER (G) | Metformin |
| 26 | METFORMIN HCL | Metformin |
| 27 | RIOMET | Metformin |
| 28 | NATEGLINIDE | Nateglinide |
| 29 | STARLIX | Nateglinide |
| 30 | PRANDIN | Repaglinide |
| 31 | ACARBOSE | Acarbose |
| 32 | PRECOSE | Acarbose |
| 33 | GLYSET | Miglitol |
| 34 | TRADJENTA | Linagliptin |
| 35 | ONGLYZA | Saxagliptin |
| 36 | JANUVIA | Sitagliptin |
| 37 | CYCLOSET | Bromocriptine |
| 38 | ACTOS | Pioglitazone |
| 39 | AVANDIA | Rosiglitazone |
| 40 | JANUMET | Dipeptidyl Peptidase-4 Inhibitor-Biguanide - Two Ingredient |
| 41 | KOMBIGLYZE XR | Dipeptidyl Peptidase-4 Inhibitor-Biguanide - Two Ingredient |
| 42 | JUVISYNC | DPP-4 Inhib-HMG CoA Reducctase Inhib Comb-2 Ingredient |
| 43 | PRANDIMET | Meglitinide-Biguanide Combination - Two Ingredient |
| 44 | GLIPIZIDE/METFORM | Sulfonylurea-Biguanide Combination - Two Ingredient |
| 45 | GLUCOVANCE | Sulfonylurea-Biguanide Combination - Two Ingredient |
| 46 | GLYBURIDE/METFORM | Sulfonylurea-Biguanide Combination - Two Ingredient |
| 47 | METAGLIP | Sulfonylurea-Biguanide Combination - Two Ingredient |
| 48 | AVANDARYL | Sulfonylurea-Thiazolidinedione Combination - Two Ingredient |
| 49 | DUETACT | Sulfonylurea-Thiazolidinedione Combination - Two Ingredient |
| 50 | ACTOPLUS MET | Thiazolidinedione-Biguanide Combination - Two Ingredient |
| 51 | ACTOPLUS MET XR | Thiazolidinedione-Biguanide Combination - Two Ingredient |
| 52 | AVANDAMET | Thiazolidinedione-Biguanide Combination - Two Ingredient |
