# Supplementary material for: Influence of peer networks on physician adoption of new drugs
Source: PLoS One. 2018 Oct 1;13(10):e0204826. doi: 10.1371/journal.pone.0204826 (PMC6166964; doi:10.1371/journal.pone.0204826)
Supplement: S2 Table — (DOCX) [file pone.0204826.s005.docx]

| **S2 Table: Distribution of number of prescriptions for the new drugs among physicians adopting them** | | | | | | | |
| --- | --- | --- | --- | --- | --- | --- | --- |
| Drug | 95^th^ %ile | 90^th^ %ile | 75^th^ %ile | **50^th^ %ile** | 25^th^ %ile | 5^th^ %ile |  |
| Dabigatran adopters | 79.0 | 52.0 | 21.5 | **7.5** | 2.8 | 1.0 |  |
| Sitagliptin adopters | 94.9 | 65.7 | 33.7 | **13.3** | 4.4 | 1.0 |  |
| Aliskiren adopters | 66.8 | 40.5 | 17.4 | **7.1** | 2.6 | 1.1 |  |
