# Supplementary material for: Influence of peer networks on physician adoption of new drugs
Source: PLoS One. 2018 Oct 1;13(10):e0204826. doi: 10.1371/journal.pone.0204826 (PMC6166964; doi:10.1371/journal.pone.0204826)
Supplement: S3 Table — Data sources: QuintilesIMS, HCOS; XPonent; AMA Masterfile 1 age of physician in 2010. (DOCX) [file pone.0204826.s006.docx]

**S3 Table: Characteristics of anticoagulant prescriber cohort and comparisons by adoption vs. non-adoption of new drug (dabigatran)**

|  | Overall | Dabigatran adopters | Dabigatran non-adopters | P-value |
| --- | --- | --- | --- | --- |
| N | 7785 | 1965 | 5820 |  |
|  |  |  |  |  |
| Mean age (years) ± SD^1^ | 50.6 ± 10.5 | 51.7 ± 9.3 | 50.2 ± 10.8 | < 0.0001 |
| Age group |  |  |  |  |
| < 35years | 652 (8.4) | 78 (4.0) | 574 (9.9) | < 0.0001 |
| 36-45 | 1914 (24.6) | 450 (22.9) | 1464 (25.2) |  |
| 46-55 | 2566 (33.0) | 697 (35.5) | 1869 (32.1) |  |
| 56+ | 2653 (34.1) | 740 (37.7) | 1913 (32.9) |  |
| # years since graduation from medical school ± SD | 23.5 ± 10.7 | 25.0 ± 9.7 | 23.0 ± 11.0 | < 0.0001 |
| Graduation year group |  |  |  |  |
| <10 | 827 (10.6) | 118 (6.0) | 709 (12.2) | < 0.0001 |
| 10-19 | 2043 (26.2) | 462 (23.5) | 1581 (27.2) |  |
| 20-29 | 2576 (33.1) | 726 (36.9) | 1850 (31.8) |  |
| 30+ | 2339 (30.0) | 659 (33.5) | 1680 (28.9) |  |
| % female | 24.8 % | 13.6 % | 28.5 % | < 0.0001 |
| Primary Specialty |  |  |  |  |
| Cardiology | 1042 (13.4) | 765 (38.9) | 277 (4.8) | < 0.0001 |
| PCP | 5579 (71.7) | 1133 (57.7) | 4446 (76.4) |  |
| Other Physicians | 1164 (15.0) | 67 (3.4) | 1097 (18.8) |  |
| Has >1 medical group affiliation (%) | 5854 (75.2) | 1550 (78.9) | 4304 (74.0) | < 0.0001 |
| Has > 1 hospital affiliation (%) | 7032 (90.3) | 1833 (93.3) | 5199 (89.3) | < 0.0001 |
| Total prescription AC volume  Mean (median) ± SD  10/2010-12/2011 | 143.0 (99.3) ± 149.2 | 253.6 (217.2) ± 178.4 | 105.7 (71.3) ± 116.3 | < 0.0001 |
| Payer mix |  |  |  |  |
| Cash | 3.6 % ± 5.5 % | 3.4 % ± 4.1 % | 3.6 % ± 5.9 % | 0.1668 |
| Commercial | 50.0 % ± 21.2 % | 51.6 % ± 15.1 % | 49.4 % ± 22.9 % | 0.0001 |
| Medicaid fee-for-service | 5.5 % ± 12.1 % | 2.7 % ± 5.2 % | 6.4 % ± 13.5 % | < 0.0001 |
| Medicare | 41.0 % ± 19.7 % | 42.3 % ± 14.4 % | 40.5 % ± 21.2 % | 0.0004 |
| Patient age mix |  |  |  |  |
| 0-64 | 37.3 % ± 23.2 % | 28.7 % ± 14.7 % | 40.2 % ± 24.7 % | <0.0001 |
| 65-74 | 22.5 % ± 15.5 % | 23.9 % ± 10.6 % | 22.0 % ± 16.8 % | <0.0001 |
| 75-84 | 26.2 % ± 16.9 % | 31.1 % ± 11.8 % | 24.5 % ± 18.0 % | <0.0001 |
| 85+ | 14.1 % ± 13.8 % | 16.4 % ± 10.4 % | 13.3 % ± 14.6 % | <0.0001 |
| Location |  |  |  | 0.2138 |
| Rural | 910 (11.7) | 245 (12.5) | 665 (11.4) |  |
| Metropolitan | 6875 (88.3) | 1720 (87.5) | 5155 (88.6) |  |
| Medical school location |  |  |  | 0.3704 |
| US | 6090 (78.2) | 1523 (77.5) | 4567 (78.5) |  |
| Foreign | 1695 (21.8) | 442 (22.5) | 1253 (21.5) |  |
| Medical school ranking |  |  |  | 0.0005 |
| Top 20 | 769 (9.9) | 234 (11.9) | 535 (9.2) |  |
| Non-Top 20 | 7016 (90.1) | 1731 (88.1) | 5285 (90.8) |  |
| HRR region |  |  |  | <0.0001 |
| Allentown | 690 (8.9) | 124 (6.3) | 566 (9.7) |  |
| Altoona | 153 (2.0) | 52 (2.6) | 101 (1.7) |  |
| Danville | 309 (4.0) | 50 (2.5) | 259 (4.5) |  |
| Erie | 339 (4.4) | 93 (4.7) | 246 (4.2) |  |
| Harrisburg | 612 (7.9) | 115 (5.9) | 497 (8.5) |  |
| Johnstown | 131 (1.7) | 53 (2.7) | 78 (1.3) |  |
| Lancaster | 375 (4.8) | 67 (3.4) | 308 (5.3) |  |
| Philadelphia | 2409 (30.9) | 526 (26.8) | 1883 (32.4) |  |
| Pittsburgh | 1657 (21.3) | 568 (28.9) | 1089 (18.7) |  |
| Reading | 337 (4.3) | 80 (4.1) | 257 (4.4) |  |
| Sayre | 72 (0.9) | 24 (1.2) | 48 (0.8) |  |
| Scranton | 193 (2.5) | 63 (3.2) | 130 (2.2) |  |
| Wilkes-Barre | 166 (2.1) | 44 (2.2) | 122 (2.1) |  |
| York | 233 (3.0) | 67 (3.4) | 166 (2.9) |  |
| Non-PA HRR | 109 (1.4) | 39 (2.0) | 70 (1.2) |  |
