# Supplementary material for: Influence of peer networks on physician adoption of new drugs
Source: PLoS One. 2018 Oct 1;13(10):e0204826. doi: 10.1371/journal.pone.0204826 (PMC6166964; doi:10.1371/journal.pone.0204826)
Supplement: S4 Table — Data sources: QuintilesIMS, HCOS; XPonent; AMA Masterfile * Age in 2007. (DOCX) [file pone.0204826.s007.docx]

**S4 Table: Characteristics of anti-diabetes medication prescriber cohort and comparisons by adoption vs. non-adoption of new drug (sitagliptin)**

|  | Overall | Sitagliptin adopters | Sitagliptin non-adopters | p-value |
| --- | --- | --- | --- | --- |
| N | 8257 | 2031 | 6226 |  |
|  |  |  |  |  |
| Mean age (years) ± SD* | 48.7 ± 10.0 | 49.1 ± 9.2 | 48.5 ± 10.2 | 0.0466 |
| Age group |  |  |  |  |
| < 35 | 742 (9.0) | 121 (6.0) | 621 (10.0) | <0.0001 |
| 36-45 | 2562 (31.0) | 633 (31.2) | 1929 (31.0) |  |
| 46-55 | 2768 (33.5) | 759 (37.4) | 2009 (32.3) |  |
| 56+ | 2185 (26.5) | 518 (25.5) | 1667 (26.8) |  |
| Graduation year (years) ± SD | 22.0 ± 10.2 | 22.5 ± 9.5 | 21.8 ± 10.5 | 0.0089 |
| Graduation year group |  |  |  | < 0.0001 |
| <10 | 1044 (12.6) | 178 (8.8) | 866 (13.9) |  |
| 10-19 | 2362 (28.6) | 594 (29.2) | 1768 (28.4) |  |
| 20-29 | 2943 (35.6) | 794 (39.1) | 2149 (34.5) |  |
| 30+ | 1908 (23.1) | 465 (22.9) | 1443 (23.2) |  |
| % female | 27.0 % | 22.7 % | 28.3 % | < 0.0001 |
| Specialty |  |  |  | < 0.0001 |
| Endocrinology | 274 (3.3) | 149 (7.3) | 125 (2.0) |  |
| PCP | 5748 (69.6) | 1809 (89.1) | 3939 (63.3) |  |
| Other Physicians | 2235 (27.1) | 73 (3.6) | 2162 (34.7) |  |
| Has medical group affiliation (%) | 5862 (71.0) | 1553 (76.5) | 4309 (69.2) | <0.0001 |
| Has hospital affiliation (%) | 7446 (90.2) | 1856 (91.4) | 5590 (89.8) | 0.0355 |
| Total prescription AD volume  1/2007-12/2007  Mean (median) ± SD | 702.4 (474.7) ± 810.5 | 1402.9 (1204.1) ± 1004.6 | 473.9 (224.5) ± 574.3 | < 0.0001 |
| Payer mix |  |  |  |  |
| Cash | 4.0 % ± 7.4 % | 3.1 % ± 2.4 % | 4.2 % ± 8.4 % | < 0.0001 |
| Commercial | 57.9 % ± 23.8 % | 61.3 % ± 15.7 % | 56.8 % ± 25.8 % | < 0.0001 |
| Medicaid fee-for-service | 8.5 % ± 15.1 % | 4.9 % ± 7.5 % | 9.7 % ± 16.7 % | < 0.0001 |
| Medicare | 29.6 % ± 19.9 % | 30.6 % ± 13.1 % | 29.3 % ± 21.6 % | 0.0080 |
| Patient age mix |  |  |  |  |
| 0-64 | 54.9 % ± 24.7 % | 51.1 % ± 14.8 % | 56.2 % ± 27.0 % | <0.0001 |
| 65-74 | 22.5% ± 16.6 % | 24.7 % ± 8.6 % | 21.8 % ± 18.5 % | <0.0001 |
| 75-84 | 16.7 % ± 15.7 % | 18.1 % ± 9.2 % | 16.3 % ± 17.2 % | <0.0001 |
| 85+ | 5.9 % ± 9.5 % | 6.1 % ± 5.1 % | 5.8 % ± 10.6 % | 0.2061 |
| Location |  |  |  | <0.0001 |
| Rural | 914 (11.1) | 283 (13.9) | 631 (10.1) |  |
| Metropolitan | 7343 (88.9) | 1748 (86.1) | 5595 (89.9) |  |
| Medical school location |  |  |  | 0.0824 |
| US | 6489 (78.6) | 1624 (80.0) | 4865 (78.1) |  |
| Foreign | 1768 (21.4) | 407 (20.0) | 1361 (21.9) |  |
| Medical school ranking |  |  |  | <0.0001 |
| Top 20 | 880 (10.7) | 140 (6.9) | 740 (11.9) |  |
| Non-Top 20 | 7377 (89.3) | 1891 (93.1) | 5486 (88.1) |  |
| HRR region |  |  |  | <0.0001 |
| Allentown | 692 (8.4) | 230 (11.3) | 462 (7.4) |  |
| Altoona | 151 (1.8) | 45 (2.2) | 106 (1.7) |  |
| Danville | 285 (3.5) | 67 (3.3) | 218 (3.5) |  |
| Erie | 339 (4.1) | 81 (4.0) | 258 (4.1) |  |
| Harrisburg | 596 (7.2) | 151 (7.4) | 445 (7.1) |  |
| Johnstown | 124 (1.5) | 33 (1.6) | 91 (1.5) |  |
| Lancaster | 373 (4.5) | 79 (3.9) | 294 (4.7) |  |
| Philadelphia | 2808 (34.0) | 606 (29.8) | 2202 (35.4) |  |
| Pittsburgh | 1785 (21.6) | 404 (19.9) | 1381 (22.2) |  |
| Reading | 337 (4.1) | 109 (5.4) | 228 (3.7) |  |
| Sayre | 78 (0.9) | 18 (0.9) | 60 (1.0) |  |
| Scranton | 192 (2.3) | 73 (3.6) | 119 (1.9) |  |
| Wilkes-Barre | 158 (1.9) | 59 (2.9) | 99 (1.6) |  |
| York | 235 (2.8) | 44 (2.2) | 191 (3.1) |  |
| Non-PA HRR | 104 (1.3) | 32 (1.6) | 72 (1.2) |  |
