# Supplementary material for: Influence of peer networks on physician adoption of new drugs
Source: PLoS One. 2018 Oct 1;13(10):e0204826. doi: 10.1371/journal.pone.0204826 (PMC6166964; doi:10.1371/journal.pone.0204826)
Supplement: S5 Table — Data sources: QuintilesIMS, HCOS; XPonent; AMA Masterfile. *Age in 2007. (DOCX) [file pone.0204826.s008.docx]

**S5 Table: Characteristics of antihypertensive prescriber cohort and comparisons by adoption vs. non-adoption of new drug (aliskiren)**

|  | Overall | Aliskiren adopters | Aliskiren non-adopters | p-value |
| --- | --- | --- | --- | --- |
| N | 9974 | 825 | 9149 |  |
| Mean age (years) ± SD* | 49.4 ± 10.1 | 50.6 ± 8.9 | 49.3 ± 10.2 | 0.0004 |
| Age group |  |  |  | < 0.0001 |
| < 35 | 910 (9.1) | 36 (4.4) | 874 (9.6) |  |
| 36-45 | 2622 (26.3) | 198 (24.0) | 2424 (26.5) |  |
| 46-55 | 3724 (37.3) | 356 (43.2) | 3368 (36.8) |  |
| 56+ | 2718 (27.3) | 235 (28.5) | 2483 (27.1) |  |
| Graduation year (years) ± SD | 22.3 ± 10.4 | 23.7 ± 9.2 | 22.2 ± 10.5 | < 0.0001 |
| Graduation year group |  |  |  | < 0.0001 |
| <10 | 1244 (12.5) | 59 (7.2) | 1185 (13.0) |  |
| 10-19 | 2732 (27.4) | 200 (24.2) | 2532 (27.7) |  |
| 20-29 | 3581 (35.9) | 349 (42.3) | 3232 (35.3) |  |
| 30+ | 2417 (24.2) | 217 (26.3) | 2200 (24.0) |  |
| % female | 24.8 % | 14.2 % | 25.7 % | < 0.0001 |
| Specialty |  |  |  | < 0.0001 |
| Cardiology | 1106 (11.1) | 120 (14.5) | 986 (10.8) |  |
| Nephrology | 311 (3.1) | 37 (4.5) | 274 (3.0) |  |
| PCP | 5959 (59.7) | 645 (78.2) | 5314 (58.1) |  |
| Other Physicians | 2598 (26.0) | 23 (2.8) | 2575 (28.1) |  |
| Has medical group affiliation (%) | 6848 (68.7) | 627 (76.0) | 6221 (68.0) | < 0.0001 |
| Has hospital affiliation (%) | 8976 (90.0) | 740 (89.7) | 8236 (90.0) | 0.7666 |
| Total prescription AH volume  3/2007-5/2008  Mean (median) ± SD | 1033.2 (671.8) ± 1141.0 | 2474.9 (2272.8) ± 1389.5 | 903.2 (545.7) ± 1020.3 | < 0.0001 |
| Payer mix |  |  |  |  |
| Cash | 4.9 % ± 8.1 % | 3.5% ± 3.2 % | 5.0 % ± 8.4 % | < 0.0001 |
| Commercial | 60.4 % ± 21.4 % | 60.8 % ± 14.7 % | 60.4 % ± 21.9 % | 0.5397 |
| Medicaid fee-for-service | 6.7 % ± 12.4 % | 4.2 % ± 6.2 % | 6.9 % ± 12.8 % | < 0.0001 |
| Medicare | 28.0 % ± 17.3 % | 31.4 % ± 12.5 % | 27.7 % ± 17.6 % | < 0.0001 |
| Patient age mix |  |  |  |  |
| 0-64 | 55.0 % ± 22.3 % | 50.0 % ± 13.8 % | 55.5 % ± 22.8 % | < 0.0001 |
| 65-74 | 20.2 % ± 13.7 % | 21.6 % ± 5.5 % | 20.1 % ± 14.2 % | 0.0021 |
| 75-84 | 16.8 % ± 13.4 % | 19.3 % ± 7.7 % | 16.5 % ± 13.8 % | < 0.0001 |
| 85+ | 8.0 % ± 9.5 % | 9.1 % ± 5.3 % | 7.9 % ± 9.8 % | 0.0003 |
| Location |  |  |  | 0.0021 |
| Rural | 1020 (10.2) | 110 (13.3) | 910 (9.9) |  |
| Metropolitan | 8954 (89.8) | 715 (86.7) | 8239 (90.1) |  |
| Medical school location |  |  |  | 0.4569 |
| US | 7827 (78.5) | 639 (77.5) | 7188 (71.6) |  |
| Foreign | 2147 (21.5) | 186 (22.5) | 1961 (21.4) |  |
| Medical school ranking |  |  |  | < 0.0001 |
| Top 20 | 1127 (11.3) | 54 (6.5) | 1073 (11.7) |  |
| Non-Top 20 | 8847 (88.7) | 771 (93.5) | 8076 (88.3) |  |
| HRR region |  |  |  | < 0.0001 |
| Allentown | 837 (8.4) | 85 (10.3) | 752 (8.2) |  |
| Altoona | 170 (1.7) | 12 (1.5) | 158 (1.7) |  |
| Danville | 340 (3.4) | 27 (3.3) | 313 (3.4) |  |
| Erie | 384 (3.9) | 31 (3.8) | 353 (3.9) |  |
| Harrisburg | 723 (7.2) | 41 (5.0) | 682 (7.5) |  |
| Johnstown | 140 (1.4) | 14 (1.7) | 126 (1.4) |  |
| Lancaster | 404 (4.1) | 26 (3.2) | 378 (4.1) |  |
| Philadelphia | 3549 (35.6) | 247 (29.9) | 3302 (36.1) |  |
| Pittsburgh | 2179 (21.8) | 184 (22.3) | 1995 (21.8) |  |
| Reading | 383 (3.8) | 61 (7.4) | 322 (3.5) |  |
| Sayre | 87 (0.9) | 8 (1.0) | 79 (0.9) |  |
| Scranton | 228 (2.3) | 37 (4.5) | 191 (2.1) |  |
| Wilkes-Barre | 190 (1.9) | 23 (2.8) | 167 (1.8) |  |
| York | 253 (2.5) | 8 (1.0) | 245 (2.7) |  |
| Non-PA HRR | 107 (1.1) | 21 (2.5) | 86 (0.9) |  |
