# Supplementary material for: Influence of peer networks on physician adoption of new drugs
Source: PLoS One. 2018 Oct 1;13(10):e0204826. doi: 10.1371/journal.pone.0204826 (PMC6166964; doi:10.1371/journal.pone.0204826)
Supplement: S6 Table — Sources: Medicare data were obtained from CMS. Medicaid data were obtained from the Pennsylvania Department of Human Services. Notes: In the anticoagulant cohort, there are 7,785 physicians meeting inclusion criteria of whom 7,522 (96.6%) had Medicare claims and 6,680 (85.8%) had Medicaid claims. We included claims submitted by those physicians to Medicare and Medicaid with dates of service between 10/1/2010 and 12/31/2011 to match the period over which we measured adoption of dabigatran. In the antidiabetic cohort, 8,257 physicians met inclusion criteria. We included claims from Medicare and Medicaid submitted by those physicians with dates of service between 1/1/2007 and 1/31/2008 (the measurement period for adoption of sitagliptin). There are 9,974 physicians meeting inclusion criteria for the antihypertensive prescriber cohort. We included claims from Medicare or Medicaid with dates of service between 3/1/2007 and 5/31/2008, the measurement period for adoption of aliskiren. (DOCX) [file pone.0204826.s009.docx]

**S6 Table: Sample size of physicians in each prescribing cohort with Medicare and/or Medicaid claims during adoption measurement period**

|  | # of physicians | Total # of claims for those physicians | Total # of patients | Mean (SD) # patients per physician |
| --- | --- | --- | --- | --- |
| Anticoagulant prescribers with Medicare claims | 7,522 (96.6%) | 7,340,465 | 669,901 | 290 (329) |
| Anticoagulant prescribers with Medicaid claims | 6,680 (85.8%) | 2,711,447 | 547,296 | 169 (261) |
| Antidiabetic prescribers with Medicare claims | 7,679 (93.0%) | 5,675,258 | 628,052 | 215 (259) |
| Antidiabetic prescribers with Medicaid claims | 6,558 (79.4%) | 2,014,312 | 444,293 | 136 (218) |
| Antihypertensive prescribers with Medicare claims | 9,164 (91.8%) | 8,173,405 | 676,533 | 273 (340) |
| Antihypertensive prescribers with Medicaid claims | 7,928 (79.5%) | 2,581,785 | 490,173 | 146 (234) |
