# Supplementary material for: Influence of peer networks on physician adoption of new drugs
Source: PLoS One. 2018 Oct 1;13(10):e0204826. doi: 10.1371/journal.pone.0204826 (PMC6166964; doi:10.1371/journal.pone.0204826)
Supplement: S7 Table — Data sources: QuintilesIMS, HCOS; XPonent; AMA Masterfile 1 Column displays the number of physicians who are not connected to any peers in a particular network. For example, physicians may lack peers in the patient-sharing network because they do not see patients with Medicaid and Medicare patients or because they did not share any patients with that source of coverage with other physicians in the prescribing cohort. (DOCX) [file pone.0204826.s010.docx]

**S7 Table: Number of physician peers to which physicians are connected in patient-sharing, medical group, hospital and training networks**

|  | Mean | Std | Min | Lower Quartile | Median | Upper Quartile | Max | N no peers in network^1^ |
| --- | --- | --- | --- | --- | --- | --- | --- | --- |
|  | **Anticoagulant prescribers, n = 7,785** | | | | | | | |
| Patient-sharing network | 287 | 253 | 0 | 119 | 202 | 381 | 2,731 | 163 |
| Medical group network | 10 | 35 | 0 | 0 | 2 | 5 | 222 | 2,777 |
| Hospital network | 166 | 148 | 0 | 58 | 122 | 246 | 883 | 763 |
| Training network | 39 | 42 | 0 | 6 | 24 | 58 | 188 | 352 |
|  | **Antidiabetic prescribers, n = 8,257** | | | | | | | |
| Patient-sharing network | 200 | 191 | 0 | 75 | 138 | 261 | 1,588 | 452 |
| Medical group network | 9 | 36 | 0 | 0 | 1 | 4 | 227 | 3,465 |
| Hospital network | 173 | 158 | 0 | 62 | 130 | 255 | 861 | 819 |
| Training network | 41 | 45 | 0 | 6 | 25 | 61 | 207 | 351 |
|  | **Anti-hypertensive prescribers, n = 9,974** | | | | | | | |
| Patient-sharing network | 344 | 316 | 0 | 127 | 248 | 475 | 3,561 | 624 |
| Medical group network | 12 | 49 | 0 | 0 | 1 | 4 | 302 | 4,262 |
| Hospital network | 204 | 187 | 0 | 63 | 161 | 291 | 1,089 | 1,007 |
| Training network | 46 | 48 | 0 | 7 | 30 | 70 | 209 | 348 |
