# Supplementary material for: Influence of peer networks on physician adoption of new drugs
Source: PLoS One. 2018 Oct 1;13(10):e0204826. doi: 10.1371/journal.pone.0204826 (PMC6166964; doi:10.1371/journal.pone.0204826)
Supplement: S8 Table — (DOCX) [file pone.0204826.s011.docx]

**S8 Table: Assessments of instrument exogeneity and relevance**

|  | **Dabigatran** | **Sitagliptin** | **Aliskiren** |
| --- | --- | --- | --- |
| *Panel A. Partial assessment of instrument exogeneity*  (overidentification test) | | | |
| Test statistic  [p-value] | 47.3  [0.031] | 37.0  [0.213] | 30.3  [0.500] |
| *Panel B. Individual assessments of instrument relevance for each network*  (first-stage *F*-statistic)  Network: | | | |
| Patient-sharing | 25.3 | 30.4 | 12.8 |
| Medical group | 5.2 | 6.3 | 3.8 |
| Hospital | 17.5 | 28.4 | 22.4 |
| Training | 2.6 | 2.7 | 1.6 |
| *Panel C. Joint assessment of instrument relevance, with medical group and training networks*  (minimum eigenvalue statistic) | | | |
| Test statistic | 5.3 | 3.4 | 2.1 |
| *Panel D. Joint assessment of instrument relevance, without medical group and training networks*  (minimum eigenvalue statistic) | | | |
| Test statistic | 51.4 | 44.3 | 17.4 |
