# Supplementary material for: Influence of peer networks on physician adoption of new drugs
Source: PLoS One. 2018 Oct 1;13(10):e0204826. doi: 10.1371/journal.pone.0204826 (PMC6166964; doi:10.1371/journal.pone.0204826)
Supplement: S9 Table — Data sources: QuintilesIMS, HCOS; XPonent; AMA Masterfile *In the analytical dataset, the unit is physician. For categorical variables, we obtain the proportion of each type (e.g., proportion of peers who are female) for each physician. For the continuous variables, we use the average percentage of pay type (e.g., Medicare) of all peers for each physician. Table shows the proportion of physicians in each network with that characteristic. (DOCX) [file pone.0204826.s012.docx]

**S9 Table: Peer characteristics used as instruments in each network**

|  | AC | AD | AH |
| --- | --- | --- | --- |
| **Patient sharing network** | N=7622 | N=7805 | N=9350 |
| Peers % graduation year group |  |  |  |
| <10 | 0.08 ± 0.06 | 0.09 ± 0.07 | 0.09 ± 0.05 |
| 10-19 | 0.25 ± 0.09 | 0.28 ± 0.11 | 0.27 ± 0.08 |
| 20-29 | 0.35 ± 0.09 | 0.38 ± 0.11 | 0.40 ± 0.08 |
| 30+ | 0.32 ± 0.10 | 0.25 ± 0.11 | 0.25 ± 0.10 |
| Peers % female | 0.16 ± 0.08 | 0.18 ± 0.10 | 0.16 ± 0.07 |
| Peers % metropolitan | 0.89 ± 0.23 | 0.89 ± 0.24 | 0.91 ± 0.22 |
| Peers % US Medical school | 0.77 ± 0.12 | 0.77 ± 0.13 | 0.77 ± 0.12 |
| Peers % top 20 school | 0.11 ± 0.08 | 0.11 ± 0.08 | 0.13 ± 0.08 |
| Peers average % cash+Medicaid | 0.08 ± 0.04 | 0.12 ± 0.06 | 0.11 ± 0.04 |
| Peers average % patient mix |  |  |  |
| 0-64 | 0.35 ± 0.05 | 0.52 ± 0.08 | 0.52 ± 0.06 |
| 65-84 | 0.51 ± 0.04 | 0.42 ± 0.07 | 0.40 ± 0.05 |
| 85+ | 0.14 ± 0.03 | 0.06 ± 0.03 | 0.08 ± 0.02 |
| **Medical group network** | N=5008 | N=4792 | N=5712 |
| Peers % graduation year group |  |  |  |
| <10 | 0.10 ± 0.20 | 0.14 ± 0.24 | 0.14 ± 0.23 |
| 10-19 | 0.28 ± 0.29 | 0.31 ± 0.31 | 0.30 ± 0.29 |
| 20-29 | 0.35 ± 0.31 | 0.36 ± 0.32 | 0.36 ± 0.31 |
| 30+ | 0.27 ± 0.29 | 0.19 ± 0.26 | 0.20 ± 0.26 |
| Peers % female | 0.26 ± 0.30 | 0.28 ± 0.30 | 0.26 ± 0.29 |
| Peers % metropolitan | 0.90 ± 0.29 | 0.91 ± 0.27 | 0.92 ± 0.26 |
| Peers % US Medical school | 0.83 ± 0.26 | 0.84 ± 0.27 | 0.83 ± 0.26 |
| Peers % top 20 school | 0.11 ± 0.20 | 0.11 ± 0.21 | 0.12 ± 0.21 |
| Peers average % cash+Medicaid | 0.08 ± 0.08 | 0.11 ± 0.10 | 0.10 ± 0.09 |
| Peers average % patient mix |  |  |  |
| 0-64 | 0.37 ± 0.16 | 0.55 ± 0.16 | 0.54 ± 0.14 |
| 65-84 | 0.49 ± 0.14 | 0.39 ± 0.15 | 0.38 ± 0.12 |
| 85+ | 0.14 ± 0.09 | 0.06 ± 0.05 | 0.08 ± 0.06 |
| **Hospital network** | N=7022 | N=7438 | N=8967 |
| Peers % graduation year group |  |  |  |
| <10 | 0.08 ± 0.05 | 0.11 ± 0.05 | 0.11 ± 0.05 |
| 10-19 | 0.26 ± 0.07 | 0.29 ± 0.07 | 0.28 ± 0.06 |
| 20-29 | 0.35 ± 0.07 | 0.37 ± 0.07 | 0.37 ± 0.07 |
| 30+ | 0.31 ± 0.08 | 0.23 ± 0.08 | 0.24 ± 0.08 |
| Peers % female | 0.23 ± 0.07 | 0.25 ± 0.07 | 0.23 ± 0.06 |
| Peers % metropolitan | 0.89 ± 0.24 | 0.89 ± 0.25 | 0.90 ± 0.23 |
| Peers % US Medical school | 0.78 ± 0.10 | 0.78 ± 0.10 | 0.79 ± 0.10 |
| Peers % top 20 school | 0.10 ± 0.08 | 0.11 ± 0.08 | 0.12 ± 0.08 |
| Peers average % cash+Medicaid | 0.09 ± 0.03 | 0.12 ± 0.04 | 0.11 ± 0.03 |
| Peers average % patient mix |  |  |  |
| 0-64 | 0.37 ± 0.05 | 0.55 ± 0.05 | 0.54 ± 0.05 |
| 65-84 | 0.49 ± 0.03 | 0.39 ± 0.04 | 0.38 ± 0.04 |
| 85+ | 0.14 ± 0.03 | 0.06 ± 0.02 | 0.08 ± 0.02 |
| **Training network** | N=7433 | N=7906 | N=9626 |
| Peers % female | 0.25 ± 0.21 | 0.27 ± 0.21 | 0.24 ± 0.20 |
| Peers % metropolitan | 0.89 ± 0.13 | 0.90 ± 0.13 | 0.90 ± 0.12 |
| Peers average % cash+Medicaid | 0.09 ± 0.06 | 0.12 ± 0.08 | 0.11 ± 0.06 |
| Peers average % patient mix |  |  |  |
| 0-64 | 0.37 ± 0.10 | 0.55 ± 0.10 | 0.55 ± 0.09 |
| 65-84 | 0.49 ± 0.09 | 0.39 ± 0.09 | 0.37 ± 0.08 |
| 85+ | 0.15 ± 0.06 | 0.06 ± 0.04 | 0.08 ± 0.04 |
