# Supplementary material for: Influence of peer networks on physician adoption of new drugs
Source: PLoS One. 2018 Oct 1;13(10):e0204826. doi: 10.1371/journal.pone.0204826 (PMC6166964; doi:10.1371/journal.pone.0204826)
Supplement: S10 Table — Primary analysis showing all covariates. Data sources: QuintilesIMS, HCOS; XPonent; AMA Masterfile Notes: Robust standard errors in parentheses *** p<0.001, ** p<0.01, * p<0.05 ahigh-volume = total prescribing volume in the category above the median. (DOCX) [file pone.0204826.s013.docx]

**S10 Table: Estimates of peer effects and covariates on adoption of new drugs from instrumental variables regression. *Primary analysis showing all covariates.***

| Variable | Anticoagulant cohort for dabigatran adoption (n = 7,785) | Antidiabetes cohort for sitagliptin adoption (n = 8,257) | Antihypertensive cohort for aliskiren adoption  (n= 9,974) |
| --- | --- | --- | --- |
| Patient-sharing network | 0.590*** (0.150) | 0.832*** (0.151) | 0.784**(0.293) |
| Medical group network | 0.185 (0.132) | 0.162 (0.140) | 0.335 (0.175) |
| Hospital network | 0.319 (0.188) | 0.376 (0.211) | 0.367 (0.261) |
| Training network | 0.0649 (0.187) | 0.0408 (0.225) | 0.0457 (0.310) |
| % high-volume prescribers^a^ | 0.201*** (0.0101) | 0.328*** (0.0110) | 0.128*** (0.00664) |
| fraction of patient-sharing peers who are high-volume prescribers | -0.262*** (0.0992) | -0.245*** (0.0657) | -0.100 (0.0531) |
| fraction of medical group peers who are high-volume prescribers | -0.0419 (0.0328) | -0.0867 (0.0561) | -0.0452 (0.0307) |
| fraction of hospital peers who are high-volume prescribers | -0.0793 (0.0920) | -0.194 (0.122) | -0.0623 (0.0616) |
| fraction of training peers who are high-volume prescribers | 0.00450 (0.0651) | -0.0147 (0.0932) | -0.0139 (0.0565) |
| Physician sex = female | -0.0443***  (0.00962) | -0.0252**  (0.00959) | -0.0268***  (0.00593) |
| Medical school graduation (<10 years = reference category) |  |  |  |
| 10-19 | -0.0185  (0.0151) | 0.00522  (0.0152) | -0.000151  (0.00932) |
| 20-29 | -0.0271  (0.0161) | 0.00394  (0.0165) | 0.0140  (0.0111) |
| 30+ | -0.0370*  (0.0168) | -0.00730  (0.0179) | 0.00835  (0.0128) |
| Relevant Physician Specialty (PCP = reference category; Cardiologist for anticoagulant and antihypertensive cohorts; endocrinologist for antidiabetic cohort) | 0.384***  (0.0217) | 0.258***  (0.0290) | 0.0190  (0.0131) |
| Nephrology |  |  | 0.0684*  (0.0270) |
| Other specialty (e.g., surgeon) | -0.0594***  (0.0124) | -0.0475***  (0.0103) | -0.0227***  (0.00678) |
| Practices in metropolitan area | -0.00955  (0.0172) | 0.00945  (0.0180) | 0.00660  (0.0138) |
| Graduated from US Medical School | -0.00352  (0.0110) | -0.00353  (0.0105) | -0.00627  (0.00708) |
| Graduated from top 20 US Medical School | 0.00389  (0.0158) | -0.0246  (0.0153) | -0.0127  (0.00886) |
| % of prescriptions paid for by Medicaid or uninsured | -0.0368  (0.0255) | -0.118***  (0.0171) | -0.0230*  (0.0107) |
| % of prescriptions filled by patients age 65-84 (reference category = % filled by patients <65 years old) | 0.122***  (0.0186) | 0.0183  (0.0126) | -0.00295  (0.00843) |
| % prescriptions filled by patients >84 years | 0.130***  (0.0301) | -0.0201  (0.0262) | 0.00693  (0.0159) |
| No peers in patient-sharing network | 0.00409  (0.0499) | 0.189***  (0.0463) | 0.0597*  (0.0286) |
| No peers in medical group network | 0.0400*  (0.0165) | -0.0114  (0.0198) | 0.0130  (0.0116) |
| No peers in hospital network | 0.0137  (0.0339) | -0.0421  (0.0510) | 0.0125  (0.0345) |
| No peers in training network | 0.00664  (0.0240) | 0.0188  (0.0278) | -0.0123  (0.0185) |
| Fraction of peers in relevant specialty (e.g., cardio, endocrine) in patient-sharing network | -0.262**  (0.0998) | -0.113  (0.140) | 0.0706*  (0.0339) |
| Fraction of peers who are nephrologists in patient-sharing network |  |  | -0.0506  (0.0835) |
| Fraction of peers who are in other specialties in patient-sharing network | 0.183*  (0.0721) | 0.361***  (0.0545) | 0.0754*  (0.0362) |
| Fraction of peers in relevant specialty (e.g., cardio, endocrine) in medical group network | 0.0552  (0.0775) | 0.0305  (0.0600) | -0.0144  (0.0181) |
| Fraction of peers who are nephrologists in medical group network |  |  | -0.00525  (0.0362) |
| Fraction of peers who are in other specialties in medical group network | 0.00699  (0.0227) | -0.0489*  (0.0212) | 0.0202  (0.0134) |
| Fraction of peers in relevant specialty (e.g., cardio, endocrine) in hospital network | -0.169  (0.107) | -0.108  (0.199) | 0.0236  (0.0530) |
| Fraction of peers who are nephrologists in hospital network |  |  | -0.0499  (0.127) |
| Fraction of peers who are in other specialties in hospital network | 0.0503  (0.0649) | -0.0843  (0.0644) | -0.00361  (0.0460) |
| Fraction of peers in relevant specialty (e.g., cardio, endocrine) in training network | -0.00792  (0.0877) | 0.0328  (0.0697) | 0.0187  (0.0248) |
| Fraction of peers who are nephrologists in training network |  |  | -0.0878  (0.0521) |
| Fraction of peers who are in other specialties in training network | 0.0469  (0.0301) | -0.00495  (0.0277) | -0.0229  (0.0178) |
| HRR2 (reference category = Philadelphia) | -0.0123  (0.0165) | 0.0354  (0.0212) | 0.0174  (0.0118) |
| HRR3 | 0.0192  (0.0391) | 0.0567  (0.0386) | 0.0423  (0.0261) |
| HRR4 | 0.0109  (0.0307) | 0.0339  (0.0311) | 0.0324  (0.0189) |
| HRR5 | 0.0539  (0.0304) | 0.0245  (0.0265) | 0.0415  (0.0219) |
| HRR6 | -0.00881  (0.0194) | 0.0198  (0.0198) | 0.0404*  (0.0167) |
| HRR7 | 0.0388  (0.0442) | 0.0428  (0.0440) | 0.0363  (0.0299) |
| HRR8 | -0.0109  (0.0243) | 0.0138  (0.0249) | 0.0340*  (0.0165) |
| HRR9 | 0.0402*  (0.0189) | 0.0191  (0.0163) | 0.0228*  (0.00956) |
| HRR10 | -0.0251  (0.0220) | 0.00819  (0.0256) | -0.0244  (0.0235) |
| HRR11 | 0.0189  (0.0545) | 0.0124  (0.0488) | 0.00957  (0.0355) |
| HRR12 | 0.0289  (0.0364) | 0.00240  (0.0348) | 0.0273  (0.0303) |
| HRR13 | 0.0305  (0.0375) | 0.0102  (0.0350) | 0.0256  (0.0243) |
| HRR14 | 0.0417  (0.0356) | 0.0604  (0.0315) | 0.0387*  (0.0184) |
| Non-PA HRRs | 0.0339  (0.0432) | 0.00964  (0.0427) | 0.0344  (0.0429) |
| R-squared | 0.326 | 0.279 | 0.111 |
