# Supplementary material for: Influence of peer networks on physician adoption of new drugs
Source: PLoS One. 2018 Oct 1;13(10):e0204826. doi: 10.1371/journal.pone.0204826 (PMC6166964; doi:10.1371/journal.pone.0204826)
Supplement: S11 Table — Data sources: QuintilesIMS, HCOS; XPonent; AMA Masterfile Notes: Robust standard errors in parentheses *** p<0.001, ** p<0.01, * p<0.05. (DOCX) [file pone.0204826.s014.docx]

**S11 Table: Estimates of peer effects on adoption of new drugs from linear model without instrumental variables.**

| Variable | Anticoagulant cohort for dabigatran adoption (n = 7,785) | Antidiabetes cohort for sitagliptin adoption  (n = 8,257) | Antihypertensive cohort for aliskiren adoption  (n= 9,974) |
| --- | --- | --- | --- |
| Patient-sharing network | 0.447*** (0.0603) | 0.486*** (0.0539) | 0.493*** (0.0737) |
| Medical group network | 0.0934*** (0.0266) | 0.167*** (0.0244) | 0.210*** (0.0286) |
| Hospital network | 0.0862 (0.0798) | 0.0799 (0.0824) | 0.0650 (0.0990) |
| Training network | 0.0205(0.0302) | 0.0801** (0.0292) | 0.000408 (0.0279) |
