# Supplementary material for: Influence of peer networks on physician adoption of new drugs
Source: PLoS One. 2018 Oct 1;13(10):e0204826. doi: 10.1371/journal.pone.0204826 (PMC6166964; doi:10.1371/journal.pone.0204826)
Supplement: S12 Table — Alternate specification of own and peer adoption (using> = 1 and 15). Data sources: QuintilesIMS, HCOS; XPonent; AMA Masterfile Notes: Robust standard errors in parentheses *** p<0.001, ** p<0.01, * p<0.05. (DOCX) [file pone.0204826.s015.docx]

**S12 Table: Estimates of peer effects and covariates on adoption of new drugs. *Alternate specification of own and peer adoption (using>=1 and 15)***

| Variable | Anticoagulant cohort for dabigatran adoption (n = 7,785) | Antidiabetes cohort for sitagliptin adoption  (n = 8,257) | Antihypertensive cohort for aliskiren adoption  (n= 9,974) |
| --- | --- | --- | --- |
|  | ***adoption defined >=1 prescription*** | | |
| Patient-sharing network | 0.956*** (0.174) | 0.877*** (0.125) | 0.782*** (0.207) |
| Medical group network | 0.319** (0.109) | 0.328*** (0.092) | 0.427** (0.152) |
| Hospital network | -0.0363 (0.189) | 0.226 (0.161) | 0.199 (0.232) |
| Training network | 0.358* (0.158) | -0.216 (0.160) | 0.392 (0.287) |
|  | ***adoption defined as >=median*** | | |
| Patient-sharing network | 0.590*** (0.150) | 0.832*** (0.151) | 0.784**(0.293) |
| Medical group network | 0.185 (0.132) | 0.162 (0.140) | 0.335 (0.175) |
| Hospital network | 0.319 (0.188) | 0.376 (0.211) | 0.367 (0.261) |
| Training network | 0.0649 (0.187) | 0.0408 (0.225) | 0.0457 (0.310) |
|  | ***adoption defined as >=15 prescriptions*** | | |
| Patient-sharing network | 0.238 (0.142) | 0.755*** (0.147) | 0.762* (0.322) |
| Medical group network | 0.211 (0.138) | 0.140 (0.142) | 0.461** (0.175) |
| Hospital network | 0.531* (0.247) | 0.491* (0.207) | 0.210 (0.305) |
| Training network | -0.358 (0.264) | -0.0563 (0.242) | -0.0132 (0.311) |
