# Supplementary material for: Influence of peer networks on physician adoption of new drugs
Source: PLoS One. 2018 Oct 1;13(10):e0204826. doi: 10.1371/journal.pone.0204826 (PMC6166964; doi:10.1371/journal.pone.0204826)
Supplement: S13 Table — (DOCX) [file pone.0204826.s016.docx]

**S13 Table: Distribution of aggregate social multipliers on individual adoption by drug and network**

|  | Mean | Std | Median | Lower Quartile | Upper Quartile | 10% | 90% |
| --- | --- | --- | --- | --- | --- | --- | --- |
| **Dabigatran** |  |  |  |  |  |  |  |
| Medical group network | 0.23 | 0.07 | 0.23 | 0.22 | 0.23 | 0.16 | 0.29 |
| Hospital network | 0.47 | 0.21 | 0.44 | 0.34 | 0.56 | 0.26 | 0.73 |
| Patient sharing network | 1.44 | 1.81 | 0.79 | 0.39 | 1.70 | 0.20 | 3.53 |
| Training network | 0.07 | 0.03 | 0.07 | 0.05 | 0.09 | 0.03 | 0.10 |
| **Sitagliptin** |  |  |  |  |  |  |  |
| Medical group network | 0.19 | 0.06 | 0.19 | 0.18 | 0.19 | 0.14 | 0.25 |
| Hospital network | 0.60 | 0.26 | 0.56 | 0.44 | 0.73 | 0.33 | 0.93 |
| Patient sharing network | 4.95 | 6.32 | 2.87 | 1.33 | 5.82 | 0.58 | 11.65 |
| Training network | 0.04 | 0.02 | 0.04 | 0.03 | 0.05 | 0.02 | 0.06 |
| **Aliskiren** |  |  |  |  |  |  |  |
| Medical group network | 0.50 | 0.16 | 0.50 | 0.48 | 0.50 | 0.35 | 0.65 |
| Hospital network | 0.58 | 0.26 | 0.54 | 0.40 | 0.70 | 0.32 | 0.92 |
| Patient sharing network | 3.63 | 4.70 | 2.01 | 0.91 | 4.32 | 0.39 | 8.82 |
| Training network | 0.05 | 0.02 | 0.05 | 0.04 | 0.06 | 0.02 | 0.07 |
