# Supplementary material for: Influence of peer networks on physician adoption of new drugs
Source: PLoS One. 2018 Oct 1;13(10):e0204826. doi: 10.1371/journal.pone.0204826 (PMC6166964; doi:10.1371/journal.pone.0204826)
Supplement: S1 Supporting Information — (DOCX) [file pone.0204826.s017.docx]

**Supporting Information:**

Methods and Results
S1 –13 Tables
S1 – 3 Figures

**METHODS**

**Data sources and descriptive statistics**

We obtained data and constructed physician-level measures from 5 sources all of which contained and were ultimately linked by National Provider Identifier (NPI). First, physician-level prescribing data were obtained from QuintilesIMS’s Xponent^TM^ database which directly captures >70% of all US prescriptions filled in retail pharmacies and utilizes a patented proprietary projection method to represent 100% of prescriptions filled in these outlets. This dataset has two important advantages in that it captures prescriptions reimbursed by all payers and has physician identifiers whereas other administrative data do not. We obtained data on all prescriptions dispensed in Pennsylvania between 2007-2011 for the 3 new drugs of interest plus all alternatives in the oral anticoagulant, antidiabetic, and antihypertensive classes (127 products in total). *Xponent^TM^ data were used to identify physicians eligible to adopt each of the three new drugs and to construct physician-level measures of new drug adoption.*

Second, we obtained information on several physician characteristics from the American Medical Association (AMA) Masterfile. The Masterfile obtains data on nearly all medical doctors (not just AMA members) and on approximately 93% of doctors of osteopathy upon graduation that it updates periodically. The Masterfile contains information on physician demographic characteristics (age, sex), specialty, practice setting, and training (e.g., medical school and year of graduation for each physician, residency program and year completed). *Masterfile data were used to construct training networks and to construct physician and peer characteristics included in our analyses.*

Third, we obtained 2011 information on each physician’s organizational affiliations along with information on each organization’s size (i.e., number of providers affiliated) and specialty (e.g., primary care or multispecialty group), from QuintilesIMS’s Healthcare Organizational Services (HCOS^TM^) Database. HCOS^TM^ captures physicians’ affiliations with over 29,000 practices, clinics, hospitals and integrated health systems in the US. Information on the type of affiliation (e.g. attending vs. admitting for hospitals) is also available for each physician’s organizational affiliations. *HCOS data were used to construct physician social networks at the medical group and hospital level.*

Fourth and fifth, we obtained administrative claims data for Medicaid and Medicare enrollees who account for approximately 1/3 of Pennsylvania residents. We obtained 2007-2012 data on all fee-for-service and managed care enrollees in PA’s Medicaid program through a Business Associate Agreement with the Pennsylvania Department of Human Services. We obtained 2007-2012 Medicare claims data for all enrollees in fee-for-service Medicare with Part D pharmacy benefits from the Centers for Medicare and Medicaid Services (CMS) through a Data Use Agreement. Both datasets contained physician identifiers (NPI) but only encrypted patient identifiers. *Medicaid and Medicare claims data were used to construct a patient-sharing social network among physicians in our study sample.*

All datasets used in our analyses are available for a fee and under data use agreement provisions required by each data provider. Below we provide links to instructions on how to obtain each data source.

***For Medicare and Medicaid enrollment and claims data:*** *ResDAC (Research Data Assistance Center),* <https://www.resdac.org/>

*Centers for Medicare and Medicaid Services,* <https://www.cms.gov/Research-Statistics-Data-and-Systems/Research-Statistics-Data-and-Systems.html>

***For Xponent, HCOS, and AMA masterfile data:***

*Quintile's IMS (now IQVIA),* <https://www.iqvia.com/locations/united-states>

**Physician samples**

We constructed three separate physician cohorts to measure adoption of each drug with some overlap among the cohorts for physicians meeting inclusion criteria for all three drug classes. Physician cohort construction proceeded in 4 steps and required a) identifying physicians prescribing the drug class of interest (oral anticoagulant, antidiabetic or antihypertensive medications), b) the presence of an AMA Masterfile record and a Pennsylvania practice address, c) the presence of a HCOS^TM^ record, and d) at least minimal prescribing in the drug category of interest (defined as >1 prescription per quarter) in the first 15 months after the relevant new drug was introduced. This time period was chosen to maximize the adoption measurement period available while imposing a similar measurement period for all three drugs subject to data availability. **S1-3** **Figs** show the sample construction and the number of providers excluded based on each exclusion criterion. Our final samples include 7,785 anticoagulant prescribers eligible to adopt dabigatran, 8,257 antidiabetic prescribers eligible to adopt sitagliptin, and 9,974 antihypertensive prescribers eligible to adopt aliskiren.

**New drugs of interest**

The three study drugs treat conditions with differences in prevalence and availability of substitutes, although all three were first-in-class, meaning they had novel mechanisms of action. Dabigatran is the first of a new class of oral anticoagulants to supplant oral warfarin, used for decades to prevent thromboembolic stroke in patients with atrial fibrillation. Dabigatran has comparable effectiveness to warfarin[1, 2] but does not require dose adjustment and close monitoring. Dabigatran may pose some risks (e.g., gastro-intestinal bleeding); however, it represents a significant therapeutic advance. Sitagliptin was the first in a new class of dipeptidyl-peptidase-4 (DPP-4) inhibitors that target a different physiologic pathway than have other diabetes medications. Sitagliptin has demonstrated short-term safety and effectiveness, and some potential advantages (weight loss). However, it is unclear what role DPP-4 inhibitors should play in a category of oral diabetes drugs that includes many effective, safe, and inexpensive medications. Aliskiren was the first in a new class of agents for hypertension (direct renin inhibitors) that joins two others (ACEI and ARB) inhibiting the renin-angiotensin-aldosterone system (RAAS) that are first line treatments for patients with hypertension. Aliskiren inhibits renin, the first step in the RAAS and does not increase bradykinin, the mechanism thought to lead to cough in patients on ACEIs. Long-term efficacy and safety compared to ACEIs and ARBs is unknown[3] and aliskiren is not typically used as first-line therapy.

**Adoption measure**

In our primary analysis, we define adoption as prescribing >= the median number of prescriptions for the new drug of interest in the first 15 months on the market among physicians with >=1 prescription. The medians shown in **S2 Table** below were 7 for dabigatran, 13 for sitagliptin and 7 for aliskiren among physicians writing at least one prescription for the new drug of interest. Many studies require only a single prescription to define adoption[4-6], however, that approach may lead to misclassification of physicians as adopters if they simply refilled a prescription for the new drug written by another physician. The binary measure of adoption (>=median prescriptions) is the dependent variable in the regression model and is also used to construct the measure of peer adoption (e.g., fraction of peers in each peer network adopting the new drug). In addition, we conducted a sensitivity analysis that required less and more stringent definitions of adoption (e.g., >=1 and >=15) in the first 15 months on the market. (see **Sensitivity analysis and S12 Table** for details).

**Characteristics of physician samples and associations with adoption of new drugs**

Characteristics of each of the three samples are shown in **S3-5 Tables.** A majority of physicians prescribing the drug classes of interest were primary care physicians (internal medicine or family medicine) making up 71.7% of anticoagulant, 69.6% of antidiabetic, and 60.8% of antihypertensive prescribers in the study cohorts. Relevant sub-specialists were significantly more likely to adopt. For example, cardiologists made up 13.4% of anti-coagulant prescribers, but 38.9% of adopters of dabigatran. A number of other characteristics were associated with adoption of the new drugs in univariate analyses including physician age, sex, prescribing volume, payer mix (e.g., percent of prescriptions paid for by Medicare or Medicaid), hospital referral region, and medical training (US vs. non-US medical school; top ranked medical school vs. not).

**Network construction and peer adoption measure**

We constructed 4 measures of peer adoption, one for each of the 4 types of networks: *training, medical group, hospital, and patient-sharing.* First, we constructed each of the 4 network types for each of the three prescribing cohorts. We used the network analysis library Igraph in python[7] for analyses. Once the networks were constructed and each physician’s peers in those networks were identified, we constructed measures of peer adoption following a similar approach for each type of network. One key difference across the 4 networks is that although peer adoption was unweighted by strength of the connection in the training, medical group, and hospital networks, we did apply weights to the connections in the patient-sharing network (i.e., the number of patients shared in the claims data) (see details below).

The *training network (T)* was constructed using data on institutions attended and dates of graduation from the AMA Masterfile. Two physicians were connected if they attended either the same medical school or the same residency program within +/-1 year of each other. For each physician in each of the three prescribing cohorts, we then constructed a measure of peer adoption from his/her training network (i.e., the fraction of physicians attending the same medical school or residency who adopted the new drug of interest, which we also refer to as the peer “adoption rate”).

The *medical group network (G)* was constructed using data from HCOS^TM^*.* We identified health care organizations as medical groups if their class of trade classification was outpatient center and their facility type was either a medical group or clinic. A majority of physicians in each of the prescribing cohorts had at least one medical group affiliation (**S3-5 Tables**) with some physicians having multiple medical group affiliations [mean (SD) number groups for anticoagulant prescribers =1.37 (0.66); for antidiabetic prescribers =1.36 (0.65); for antihypertensives=1.38 (0.70)]. Physician peers were identified from all medical groups with which they were affiliated. For each physician, we constructed a measure of peer adoption (adoption rate) in his/her medical group(s) equal to the fraction of peers adopting the new drug of interest in all medical groups with which a particular physician was affiliated.

The *hospital network (H)* was constructed similarly using data from HCOS^TM^. Approximately 90% of physicians in each prescribing cohort had an affiliation with at least one hospital identified as an organization with the class of trade classification equal to hospital. The mean (SD) number of hospitals with which sample physicians were affiliated was 1.95 (1.22) for anticoagulant prescribers, 1.91 (1.15) for antidiabetic prescribers, and 1.96 (1.21) for antihypertensive prescribers. For each physician, we constructed a measure of peer adoption (adoption rate) in his/her hospital(s) equal to the fraction of peers practicing in the same hospital setting who adopted the new drug of interest.

The *patient-sharing network (P)* was constructed using a previously validated approach to constructing physician social networks using administrative claims data. Barnett and colleagues developed and validated a physician network that deems two physicians to be connected if they submit claims for services delivered to the same patient in a given time period (e.g., calendar year) [8]. Comparing self-reported connections among physicians elicited through a web-based survey to connections based on shared patients in Medicare data, Barnett and colleagues found that patient-sharing measured in claims data is predictive of self-reported physician relationships for referral, information or advice. Specifically, the probability that a given physician recognizes a professional relationship with another physician increased with the number of Medicare patients shared in common, plateauing at 9-10 shared patients. The authors reported an area under the receiver-operator characteristic curve of 0.73 (95% CI 0.70-0.75).

We broaden Barnett et al’s approach to include not only Medicare patients but also Medicaid patients, as referral and advice relationships may vary depending on the health needs and demographic characteristics of patients with different sources of coverage. In other words, we identified two physicians as connected if both had claims in the Medicare carrier files for at least one unique enrollee, or if they both had claims in the Medicaid professional files for at least one unique enrollee, or had shared patients in Medicare and in Medicaid. We identified all claims submitted by sample physicians during the same period over which adoption of the new drug was measured for each cohort (depending on the date of introduction of the new drug) (**S6 Table**). We *did not* limit claims to those for patients with the particular condition or use of the drugs of interest but rather included all patients cared for by our sample physicians. Over 90% of cohort physicians billed for at least one Medicare enrollee with an average number of Medicare patients equal to 215-290 depending on the prescribing cohort. Approximately 80% of the physicians in each cohort submitted claims for one or more Medicaid enrollees with an average number of Medicaid patients equal to 136-169 depending on the prescribing cohort. Because we excluded enrollees dually eligible for Medicare and Medicaid from the Medicaid patient sample but kept them in the Medicare patient sample, these two patient groups are non-overlapping.

Unlike the other three networks in which connections were unweighted, we applied weights to the peer adoption measure in the patient-sharing network equal to the number of patients shared. Thus, the peer adoption measure (adoption rate) was equal to the fraction of peers adopting the new drug of interest with each peer weighted by the total number of Medicare and/or Medicaid patients shared.

**Descriptive statistics on network sizes**

**S7 Table** displays the distribution of network degree for the 12 networks (4 types for 3 prescribing cohorts). In each cohort, the patient-sharing network has the highest average degree, ranging from 200-344 depending on the cohort. A small share of providers (2-6%) have no peers in the patient sharing network because they did not bill Medicare or Medicaid, or because they did not share Medicare or Medicaid patients with another prescriber in the cohorts. The next largest average degree is in the hospital network, with the mean number of peers sharing a common hospital affiliation varying from 166-204, depending on the cohort. Ten percent of physicians in each cohort were not connected to any peers through a hospital affiliation either because they did not have such an affiliation or because they did not practice in the same hospital as other prescribers in the cohorts. The medical group and training networks have substantially smaller average degree than the patient-sharing and hospital networks. A larger share of physicians (36-43%) had no peers in the prescribing cohorts through shared medical group affiliation. As noted below where we describe our estimation procedure, we included an indicator variable in regression models for physicians missing peers in each type of network and who were thus missing the relevant peer adoption measure as this was the most flexible approach to handling missing information and allowed us to retain a sample of physicians with variation in practice setting and peer networks.

**Model and estimation of peer effects**

We used linear probability models to estimate the effects of peer adoption in each of the four networks (*P, G, H, T*), along with other factors, on the individual adoption outcome of each physician. Our primary analysis estimated these linear models via two-stage least squares, as described further below, which enables the use of instrumental variables in a straightforward manner. We also estimated the association between peer adoption and own adoption via a simple linear regression for comparison. Additionally, to determine whether a linear model was appropriate, we compared the estimated outcomes of adoption from a linear model to a logistic regression model and found the mean and median differences of the estimated values to be within <=0.01%. In order to allow us to easily implement instrumental variables methods using a standard statistical package we use the linear probability model.

Our models, which were estimated separately for each drug, were specified as follows:

The variables are described here in sequence. The dependent variable *y_i_* is the binary indicator of adoption. The vector *x_i_* contains individual-level covariates, which included a number of characteristics of the physicians in our analyses that have been shown to be correlated with new drug adoption. We included an indicator for whether the physician was male (reference) or female, and a categorical indicator of the number of years since they graduated from medical school (<10 years (reference), 10-19 years, 20-29 years, and >30 years). Variables for location of medical school (US or non-US) as well as medical school ranking (top 20 according to the 2011 *US News and World Report)* were also included. To account for regional variation in prescribing practices we included indicator variables for hospital referral region (HRR) of which there are 14 in Pennsylvania. A small number of physicians with practices physically in Pennsylvania were nevertheless assigned to a non-Pennsylvania HRR based on their zip code so we included a 15^th^ category for non-PA HRR. Additionally, a dichotomous variable for location of practice in a metropolitan vs. non-metropolitan area was included in the regression. Our XPonent^TM^ data contain two types of information on patients filling the prescriptions (age and payer type). Therefore we included variables for the share of a physicians’ prescriptions filled by patients aged <65 years, 65-84, or 85+ as well as the share of prescriptions paid for by Medicaid or uninsured patients, Medicare, vs. commercial insurance plans. We also included a measure of total prescribing volume in the medication class of interest during the period when adoption was measured, specified as a dichotomous variable equal to 1 if the physician had total prescribing volume above the median. Next, the variables $\overline{y}_{Pi}$ to $\overline{y}_{Ti}$ are the peer adoption rates in each network, and the variables $\overline{c}_{Pi}$ to $\overline{c}_{Ti}$ are the proportions of peers in each network in relevant specialties. The relevant specialties were cardiology for anticoagulants, endocrinology for antidiabetic drugs, and cardiology and nephrology for antihypertensives. We treated peer prescribing volume similarly by including 4 variables $\overline{v}_{Pi}$ to $\overline{v}_{Ti}$ for the fraction of peers in each network who were high-volume (above median) prescribers. The model also included indicator variables for whether a physician has no peers in each network (4 indicators, not shown in the equation). Last, the error term represents unobserved factors affecting whether physician *i* adopts the drug. The key estimates of interest from these models were the coefficients to on the peer adoption rates.

We used linear probability models to estimate the effects of peer adoption in each of the four networks (*P, G, H, T*), along with other factors, on the individual adoption outcome of each physician. Our primary analysis estimated these linear models via two-stage least squares, as described further below, which enables the use of instrumental variables in a straightforward manner. We also estimated the association between peer adoption and own adoption via a simple linear regression for comparison. Additionally, to determine whether a linear model was appropriate, we compared the estimated outcomes of adoption from a linear model to a logistic regression model and found the mean and median differences of the estimated values to be within <=0.01%. In order to allow us to easily implement instrumental variables methods using a standard statistical package we use the linear probability model.

Our models, which were estimated separately for each drug, were specified as follows:

The variables are described here in sequence. The dependent variable *y_i_* is the binary adoption measure. The vector *x_i_* contains individual-level covariates, which included a number of characteristics of the physicians in our analyses that have been shown to be correlated with new drug adoption. We included an indicator for whether the physician was male (reference) or female, and a categorical indicator of the number of years since they graduated from medical school (<10 years (reference), 10-19 years, 20-29 years, and >30 years). Variables for location of medical school (US or non-US) as well as medical school ranking (top 20 according to the 2011 *US News and World Report)* were also included. To account for regional variation in prescribing practices we included indicator variables for hospital referral region (HRR) of which there are 14 in Pennsylvania. A small number of physicians with practices physically in Pennsylvania were nevertheless assigned to a non-Pennsylvania HRR based on their zip code so we included a 15^th^ category for non-PA HRR. Additionally, a dichotomous variable for location of practice in a metropolitan vs. non-metropolitan area was included in the regression. Our XPonent^TM^ data contain two types of information on patients filling the prescriptions (age and payer type). Therefore we included variables for the share of a physicians’ prescriptions filled by patients aged <65 years, 65-84, or 85+ as well as the share of prescriptions paid for by Medicaid or uninsured patients, Medicare, vs. commercial insurance plans. We also included a measure of total prescribing volume in the medication class of interest during the period when adoption was measured, specified as a dichotomous variable equal to 1 if the physician had total prescribing volume above the median. Next, the variables $\overline{y}_{Pi}$ to $\overline{y}_{Ti}$ are the peer adoption rates in each network, and the variables $\overline{c}_{Pi}$ to $\overline{c}_{Ti}$ are the proportions of peers in each network in relevant specialties. The relevant specialties were cardiology for anticoagulants, endocrinology for antidiabetic drugs, and cardiology and nephrology for antihypertensives. We treated the prescribing volume variable similarly including 4 variables corresponding to the fraction of peers in each network that were high-volume (above median) prescribers. The model also included indicator variables for whether a physician has no peers in each network (4 indicators, not shown in the equation). Last, the error term represents unobserved factors affecting whether physician *i* adopts the drug. The key estimates of interest from these models were the coefficients to on the peer adoption rates.

There are multiple challenges to estimating peer effects using observational data [9, 10]. Many of these challenges are resolved if data on network linkages between individuals are available, as in the present study, compared with situations in which the observed “network” consists simply of a set of groups within which all members interact uniformly [11]. With data on individual linkages, it is, in concept, possible to include all characteristics of peers in the main model (not just the proportion who are specialists, as in our model, but also the proportion who are female, the proportion from a top medical school, and so on), and then use the characteristics of the *peers of peers* as instruments. Given our understanding of the study context, however, we only included the proportions of peers who were specialists and who were high volume prescribers and then used the remaining characteristics of the immediate peers as instruments. This is likely to provide more efficient estimation, so long as the other peer characteristics do not have their own direct influence on adoption. Thus, with this approach to estimation our main identifying assumption is that the means of peer characteristics (other than specialty and prescribing volume) are exogenous. Under this assumption, which can be partially assessed, the issues of simultaneous causality, correlated unobservables, and homophily are therefore addressed by using these instruments. With regard to homophily, for example, exogeneity of the instruments implies that the predicted peer adoption rates used in estimation are stripped of any common tendencies among linked individuals to adopt a new drug or not.

We estimated the models using two-stage least squares (2SLS). In this method, conceptually, a set of first-stage linear models are used to generate predicted peer adoption rates in each network. These first-stage models have the observed adoption rates (e.g., $\overline{y}_{Pi}$ for the patient-sharing network) as the dependent variables, and include individual characteristics ($x_{i}$) and the means of peer characteristics ($\overline{x}_{Pi}$, $\overline{x}_{Gi}$, $\overline{x}_{Hi}$, $\overline{x}_{Ti}$) as the independent variables. The estimated first-stage models then yield predicted peer adoption rates for each network, which are used in place of the observed adoption rates to estimate the main model in the second stage. In actuality, the first and second stages are estimated jointly using a projection matrix that contains the instruments and exogenous regressors [12].

The *exogeneity* of the instruments can be partially assessed via an overidentification test [12]. Assuming that at least one instrument is exogenous per each endogenous variable in the main model, then this test evaluates whether the other instruments are exogenous as well. One does not need to specify which instruments are assumed to be exogenous, just that there are enough such instruments. In our case, there were 25 instruments (the means of various peer characteristics, listed further below) and four endogenous variables (the peer adoption rates in each of the four networks). Hence for the test to be valid at least four of the 25 instruments must be exogenous. This *a priori* assumption that at least four instruments are exogenous is a significant and unavoidable limitation, and so we view the overidentification test as a useful but only partial assessment. On the other hand, the overidentification test also assesses the functional specification of a model (e.g., the definitions of the variables, the presence or absence of interaction terms in the model, etc.), and in this sense it can also be conservative. With these limitations in mind, we view the results in Panel A of **S8 Table** as providing an overall indication that our instruments are exogenous. For two out of the three drugs the *p*-values of the overidentification tests are well above 0.05, which means that, assuming any four instruments are exogenous, the null hypothesis that the remaining instruments are also exogenous is not rejected at the 5% level of significance. The *p*-value of 0.031 for dabigatran does raise concern, on the other hand, but the model estimates for this drug are broadly similar to those for the other drugs where the *p*-values are larger. We believe this test result may indicate other relatively minor issues with the functional specification, rather than a violation of exogeneity.

Another important concern with 2SLS estimation is the *relevance* of the instruments, meaning that the instrumental variables must provide sufficiently precise predictions for the endogenous variables. In our case, the peer mean characteristics must predict the peer adoption rates accurately. We assess this issue using two standard measures: the first-stage *F*-statistic and the minimum eigenvalue statistic [13]. The first-stage *F*-statistic assesses how well the peer adoption rate in each network is predicted individually, and the minimum eigenvalue statistic assesses how well the four adoption rates are predicted jointly. Both statistics are interpreted in terms of the amount of bias that may be present in 2SLS estimation compared to naïve ordinarly least squares (OLS) estimation. The common acceptable level for this relative bias is 10%, so that 2SLS has 90% less bias compared to OLS. Accordingly, we consider our instruments to be sufficiently predictive if the test statistics indicate that the relative bias is less than 10%. Values of the first-stage *F*-statistic greater than 10 are typically considered to indicate this, and Stock and Yogo (2005)[13] provide analogous thresholds in the minimum eigenvalue statistic for various numbers of instruments and endogenous variables.

Panels B and C in **S7 Table** present the values of these statistics for each cohort, using our main specification. For all three drugs, the first-stage *F*-statistics indicate that our instruments were sufficiently predictive of the peer adoption rates in the patient-sharing network and the hospital network, but not in the medical group network or the training network. As a consequence we do not consider the estimated peer effects in the medical group or training networks to be reliable. In the joint assessment of instrument relevance using the minimum eigenvalue statistic, the minimum acceptable value is roughly 11 (extrapolated from Table 5.1 of Stock and Yogo 2005). Hence, this statistic indicates that our instruments would not adequately predict the adoption rates jointly—i.e., for all four networks. To assess the strength of the joint prediction in just the patient-sharing and hospital networks, we then estimated models without the medical group and training networks. These models omit the instrumental variables for the medical group and training networks in the first stage and the peer adoption rates for those two networks in the second stage. Panel D reports the minimum eigenvalue statistics computed for these models. In this case, the minimum threshold in the statistic to indicate a relative bias less than 10% is 11.03 (as reported in Stata, based on Table 5.1 of Stock and Yogo 2005). The minimum eigenvalue statistic is above this value for all three drugs, indicating our instruments were sufficiently predictive for the adoption rates in the patient-sharing and hospital networks for all three drugs.

The instrumental variables used in our main specification are listed in **S9 Table**. We removed certain variables from the list of individual characteristics in **S3-5 Tables** because those variables provided little or no additional variation to help predict the peer adoption rates. For the training network, we removed the distribution of the number of years since graduation and the proportions of peers from top 20 medical schools and from US medical schools, because these variables are similar among individuals connected in the training network. We also removed the average share of prescriptions paid by Medicare as an instrument in all networks, because this is highly (negatively) correlated with the proportion of patients under 65 years old.

**Sensitivity analysis**

We conducted two sensitivity analyses as a check of the robustness of our findings to alternate specifications of the own and peer adoption measures. The first alternative was to simply require a single prescription for the new drug of interest during the first 15 months on the market. The second, more stringent alternative required >=15 prescriptions in the first 15 months. As with our main analysis, these models were estimated via 2SLS. **See S12 Table** for results.

**Computation of the aggregate social multiplier**

Our measure of the aggregate social multiplier on individual adoption is a form of Bonacich power centrality[14], where the coefficient for the effect of the peer adoption rate on the individual adoption probability (i.e., ) serves as the attenuation parameter. This measure captures the influence of a physician not only on her immediate peers, but also on her peers’ peers, etc. The social multiplier is an individual-level measure, defined separately for each network (and each drug) by the following formulas:

(for the patient-sharing network)


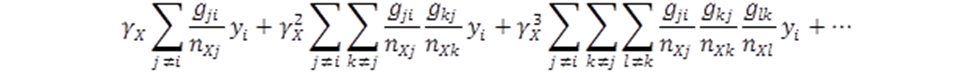


(for the medical group, hospital, and training networks respectively, where *X* = *G*, *H*, or *T*).

In the formula for the patient-sharing network, $\gamma_{P}$ is the estimated peer effect on adoption in the patient-sharing network and *w_ji_* is the weight on the link between physicians *j* and *i* based on the number of shared patients. Specifically, *w_ji_* equals the number of patients shared by physicians *j* and *i* divided by *j*’s total number of patients. This formula yields the multiplier on adoption by physician *i.* The first term in the formula is the influence on *i*’s peers, the second term is the influence on *i*’s peers’ peers, and the third term is the influence on *i*’s peers’ peers’ peers. In the formula for the other networks, $\gamma_{X}$ is the estimated peer effect on adoption in the respective networks, *g_ji_* indicates that physician *j* is linked to physician *i* in network *X*, and *n_Xj_* is physician *j*’s degree in network *X*. As with the formula for the patient-sharing network, the first term is the influence on *i*’s peers, the second term is the influence on *i*’s peers’ peers, and the third term is the influence on *i*’s peers’ peers’ peers.

To compute the multiplier we used the command *alpha_centrality* from the package *igraph* in *R*. The inputs to this command are the estimated peer effect ($\gamma_{P}$ or $\gamma_{X}$) and the network (the matrix of *w_ji_* or *g_ji_* / *n_Xj_* for all *j,i* pairs).

**RESULTS**

**Main regression results**

Below, **S10 Table** shows the full results from three 2SLS models of adoption of the three drugs of interest. Directly following, **S11 Table** shows the results for the main variables of interest from linear models estimated without the instrumental variables (i.e., simply using OLS). The results presented in **S10 Table** include the main independent variables of interest (peer adoption rates from 4 networks), which we presented and offered interpretation for in the main body of the paper (**Fig 3**). Full results are provided here for readers interested in the covariate estimates. Female physicians were roughly 3-4% less likely to adopt the new drugs of interest relative to their male counterparts after adjusting for age, specialty, peer effects, training location, and patient payer and age composition. For every 1% increase in the fraction of peers in their patient-sharing network who were high-volume prescribers physicians were 1.0-2.6% less likely to adopt the new drugs of interest. The fraction of peers in the other 3 networks who were high-volume prescriber was not associated with physician adoption. The main specialists of interest, cardiologists and endocrinologists, were more likely than primary care physicians to adopt dabigatran and sitagliptin, respectively, but cardiologists and nephrologists were no more likely than primary care physicians to adopt aliskiren. Other specialists (e.g., surgeons) were 2-6% less likely than primary care physicians to adopt dabigatran, sitagliptin, and aliskiren. For every 1% change in the share of a physicians’ prescriptions filled by older patients (age 65-84), anticoagulant prescribers were 1.2% more likely to adopt dabigatran. The age of patients filling prescriptions was not significantly associated with physician adoption of the other drugs. And the increasing share of a physician’s prescriptions paid for by Medicaid fee-for-service programs or by cash for patients without drug coverage was negatively associated with adoption of all three drugs.

In the simple regression estimates shown in **S11 Table**, the associations between peer adoption rates and own adoption are typically smaller than the peer effects estimated using instrumental variables. Unlike our main results, however, these regression estimates cannot be given a causal interpretation because they do not address the issue of simultaneous causation (i.e., the fact that each physician influences her peers just as they influence her). The quantitative differences between these OLS regression estimates and our main 2SLS instrumental variables estimates arise from two principal sources of bias. One is endogeneity, which includes simultaneity, homophily, and unmeasured confounders that are correlated among peers, as discussed in the main text. Endogeneity would tend to produce an upward bias in OLS estimates. The other source of bias is measurement error, which can occur in estimates of peer effects due to sampling noise or noise in the definition of the adoption measure. Measurement error produces a downward bias in OLS estimates, because the noise in the observed value of a variable attenuates its correlation with the outcome. Because the OLS regression estimates in **S11 Table** are smaller than the 2SLS instrumental variables estimates, this indicates that the downward measurement error bias outweighs the upward endogeneity bias. We rely on the IV approach as our primary estimation strategy because it avoids both of these biases and allows a causal interpretation of the peer effects.

**Results from sensitivity analyses for adoption measure**

Below we display the results for the main variables of interest from the sensitivity analyses we conducted altering the specification of the adoption measure. We note that the number and percent of physicians adopting the new drug changes significantly depending on the specification of this variable. The proportion of anticoagulant prescribers adopting dabigatran goes from 48.3% when adoption is defined as >=1 prescription, to 25.2% when defined as prescribing at least the median, to 16.2% when defined as prescribing >=15. The corresponding percent of antidiabetic prescribers classified as adopting sitagliptin were 49.6%, 24.6% and 22.8%; and for antihypertensives it was 16.5%, 8.3% and 4.7%. The main results on the effect of peer adoption in the patient-sharing network is stable across these different specifications of the adoption variable for the antidiabetic drug sitagliptin and the antihypertensive aliskiren and less consistent for the anticoagulant dabigatran, a finding we attribute to the differences in prescribing volume across the classes. The mean total number of prescriptions written for all anticoagulants was 143 during the adoption period, compared to a mean of 702 antidiabetic prescriptions and 1,033 antihypertensive prescriptions. Defining adoption as writing >=15 prescriptions for dabigatran is likely too restrictive for the anticoagulant category and we believe our main specification choosing the median as the cutpoint is the appropriate one.

**Additional information on multiplier illustration**

**S13 Table** shows the distribution of the social multipliers on adoption for each drug in each network (in all cases the estimated peer effect was between zero and one). The values can be interpreted as the expected number of additional adoptions that might follow from adoption by a given physician. For example, the mean of 1.44 for dabigatran in the patient-sharing network indicates that, on average, adoption by an individual physician would lead to 1.44 additional adoptions by others via this network. Importantly, these magnitudes represent the effects of marginal changes, such as adoption by an individual physician holding all else constant, as opposed to a multiplier on adoption by a large number of individuals. Hence, the multipliers are useful for identifying relatively more or less influential individuals, but they would not quantify the impact of a large-scale intervention.

The multipliers in the patient-sharing network are much larger and exhibit more variation than the multipliers in the medical group and hospital networks. In the latter two networks, physicians tend to be more equally connected with each other, and groups are more self-contained. This reduces the variance of the Bonacich power centrality. Also the attenuation parameters are smaller in those networks, which reduces the magnitude. In the patient-sharing networks for the anticoagulant and antihypertensive prescriber cohorts, on the other hand, the 75^th^ percentile multiplier is more than times larger than the 25^th^ percentile multiplier. This arises from differences across individuals in their network positions, not only in terms of degree (**S7 Table**) but also in their more distant connections which are included in the Bonacich measure.

**References:**

1. Connolly SJ, Ezekowitz MD, Yusuf S, Eikelboom J, Oldgren J, Parekh A, et al. Dabigatran versus Warfarin in Patients with Atrial Fibrillation. NEJM. 2009;361:1139-51.

2. Schneeweiss S, Gagne J, Patrick A, Choudhry N, Avorn J. Comparative efficacy and safety of new oral anticoagulants in patients with atrial fibrillation. Circulation: Cardiovasc Qual Outcomes 2012;5:480-6.

3. Norris S, Weinstein J, Peterson K, Thakurta S. Drug class review: direct renin inhibitors, angiotensin converting enzyme inhibitors, and angiotensin II receptor blockers: Drug Effectiveness Review Project, Oregon Evidence-based Practice Center, Oregon Health & Science University; 2010.

4. Iyengar R, Van de Bulte C, Valente TW. Opinion leadership and social contagion in new product diffusion Marketing Science. 2011;30(2):195-212.

5. Van den Bulte C, Lilien G. Medical innovation revisited: social contagion versus marketing effort. American Journal of Sociology. 2001;106(5):1409-35.

6. Dybdahl T, Andersen M, Sondergaard J, Kragstrup J, Kristiansen I. Does the early adopter of drugs exist? a population-based study of general practitioners' prescribing of new drugs. European Journal of Clinical Pharmacology. 2004;60(9):667-72.

7. Csardi G, Nepusz T. The igraph software package for complex network research. Inter-Journal, Complex Systems. 2006.

8. Barnett ML, Landon BE, O'Malley AJ, Keating NL, Christakis NA. Mapping physician networks with self-reported and administrative data. Health Services Research 2011;46 1592-609.

9. Manski CF. Identification of Endogenous Social Effects: The Reflection Problem. Review of Economic Studies. 1993;60(3):531-42.

10. Moffitt RA. Policy Interventions, Low-Level Equilibria, and Social Interactions. In: Durlauf S, Young H, editors. Social Dynamics2001. p. 45-82.

11. Bramoullé Y, Djebbari H, Fortin B. Identification of Peer Effects through Social Networks. Journal of Econometrics. 2009;150:41-55.

12. Wooldridge JM. Econometric analysis of cross section and panel data: MIT press; 2010.

13. Stock JH, Yogo M. Testing for weak intruments in linear IV regression. In: Donald WA, Stock JH, editors. Identification and inference for econometric models: Essays in honor of Thomas Rothenberg: Cambridge University Press; 2005.

14. Bonacich P. Power and centrality: a family of measures. American Journal of Sociology. 1987;92(5):1170-82.
